# Uncovering the mechanical secrets of the squirting cucumber

**DOI:** 10.1073/pnas.2410420121

**Published:** 2024-11-25

**Authors:** Finn Box, Derek E. Moulton, Dominic Vella, Yuvraj Bhagotra, Tristan Lowe, Alain Goriely, Chris J. Thorogood

**Affiliations:** ^a^Department of Physics and Astronomy and Manchester Center for Nonlinear Dynamics, University of Manchester, Manchester M13 9PL, United Kingdom; ^b^Mathematical Institute, University of Oxford, Oxford OX2 6GG, United Kingdom; ^c^Manchester X-ray Imaging Facility, Photon Science Institute, University of Manchester, Manchester M13 9PL, United Kingdom; ^d^University of Oxford Botanic Garden, Oxford OX1 4AZ, United Kingdom; ^e^Department of Biology, University of Oxford, Oxford OX1 3RB, United Kingdom

**Keywords:** ballistic seed dispersal, turgor pressure, mathematical models

## Abstract

Rapid movement is rare in the plant kingdom, but a prerequisite for ballistic seed dispersal. A particularly dramatic example of rapid motion in plants is the squirting cucumber (*Ecballium elaterium*) which launches its seeds explosively via a high-pressure jet. Despite intriguing scientists for centuries, the exact mechanism of seed dispersal and its effect on subsequent generations remain poorly understood. Here, through a combination of experimentation, high-speed videography, quantitative image analysis, and mathematical modeling, we develop a full mechanical description of the process. We quantify the turgor pressure driving ballistic ejection, and uncover key mechanical interactions between the fruit and stem both prior to and during seed ejection, including the unique feature that fluid is redistributed from fruit to stem prior to ejection, a developmental event that goes against the paradigm of rapid seed ejection but which is of key importance in successful dispersal for *Ecballium*. Combining modeling elements, we quantify and simulate the ballistic trajectories of seeds, which are dispersed over distances greater than 2,000 times their length. We demonstrate how together these mechanical features contribute to a nearly uniform distribution of seeds away from the parent plant. Parametric variation of key developmental events in the modeling framework indicates how a suite of adaptive features in combination drives the spatial distribution of offspring over consecutive generations, and suggests that ballistic seed dispersal has a stabilizing effect on population dynamics by reducing intraspecific competition.

## Significance

We study the remarkable seed dispersal mechanism of *Ecballium elaterium*, commonly known as the squirting cucumber, one of the most rapid motions in the plant kingdom. Despite its apparent simplicity, the specifics of the seed ejection process—combining mechanical, hydraulic, and ballistic phenomena—remain largely unexplored. By integrating experiments, high-speed videography, and advanced mathematical modeling, we uncover unique facets of this strategy, including an unusual decrease in fruit volume prior to ejection which stiffens the stem and orients the fruit to an improved angle for dispersal. Our study reveals how the delicate interaction of mechanical components contributes to dispersal efficiency, thereby influencing plant distribution and population dynamics, and offering insights into evolutionary adaptations related to explosive fruit mechanisms.

The evolution of the seed transformed life on earth. Seed plants, including flowering plants, reset the Earth-life system and drove a macroecological revolution on land (1). Seeds enable dormancy in a plant’s life cycle and colonization of new environments. Accordingly, diverse solutions to the problem of dispersing seeds have evolved, either by abiotic vectors (such as gravity, wind, or water) or biotic vectors (such as animals, including humans). Autogenic seed dispersal refers to mechanisms produced by the plant itself i.e., in the absence of external dispersal agents. This class includes some of the most remarkable feats of natural engineering found in the plant kingdom. For instance, the fruits of the deadly sandbox tree (*Hura crepitans*) (2) explode to launch disk-shaped seeds at 70 m/s to distances of up to ∼30 m (3). Other examples of explosive seed projection include the rapid coiling of capsules in *Cardamine hirsuta* (4) and *Impatiens* spp. (5, 6), and the seed catapulting in *Oxalis* sp. (7) and *Ruellia brittoniana* (Acanthaceae) (8).

A typical mechanism for explosive seed projection is a sudden morphological reconfiguration that converts stored elastic energy into the kinetic energy of projectile seeds through elastic snap-buckling, cavitation, or fracture (4–11). Another mechanism is turgor-driven ballistic autochory by which the seed or spore is forcefully ejected by the fruit or capsule as a result of turgor pressure caused by the build-up of internal hygroscopic tension, leading to explosive dehiscence (bursting) (12–14); this is frequent in the fungus kingdom (15, 16) as a means of spore dispersal and among spore plants, for example in sphagnum moss spore capsules (17). In vascular plants, however, this mechanism is rare. The berries of the mistletoe *Arceuthobium* (Loranthaceae) disperse seeds in a stream of mucilage (18, 19) following the build-up of osmotic pressure and thermogenesis-induced dehiscence (20). This shows striking parallels with the squirting cucumber, and an apparent example of convergent evolution. However, the evolutionary drivers behind ballistic seed dispersal remain unknown.

**Fig. 1.**
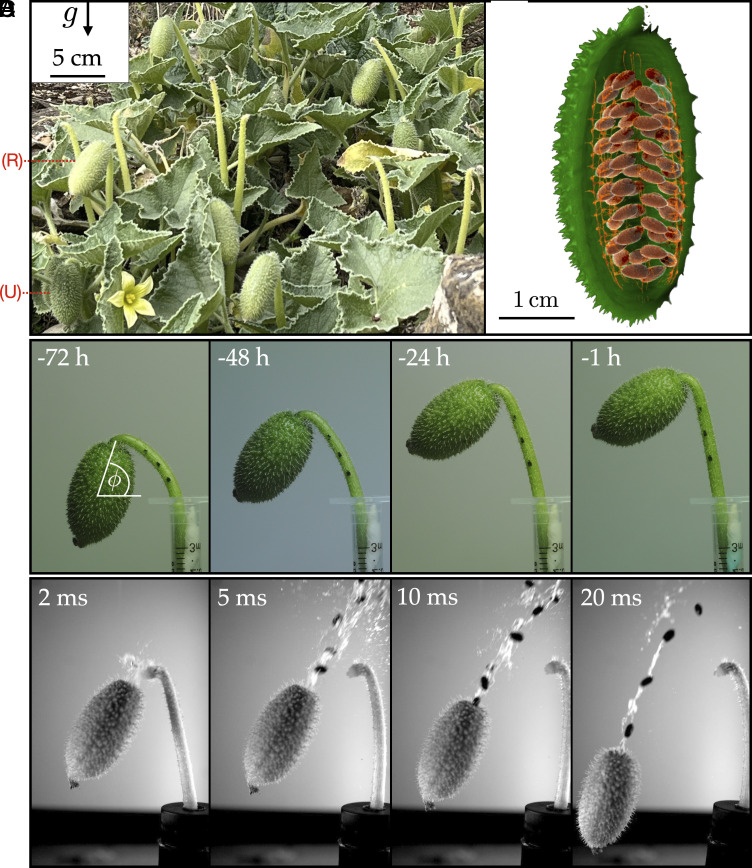
The squirting cucumber. (*A*) Images of *Ecballium elaterium* in the Oxford Botanic Garden. (R and U) indicate ripe and unripe fruits. (*B*) Computed Tomography (CT) scan of the cucumber shows how the seeds are arranged in pairs attached to four pillars located at 90 degree intervals around the long axis of the fruit. (For CT scan video see Movie S1.) (*C*) Images of fruit reorientation at t-minus 72, 48, 24, and 1 h (*Left*-to-*Right*) to launch. (*D*) Images of seed dispersal at 2, 5, 10, and 20 ms (*Left*-to-*Right*) after abscission of the stem.

The squirting cucumber *Ecballium elaterium*, shown in Fig. 1*A*, is a member of the gourd family (Cucurbitaceae) which contains about 1,000 species (including the melon, pumpkin, squash, and zucchini). Many are climbing vines with twining tendrils that wrap around and cling to slender objects they come into contact with (21, 22). *Ecballium* is unique in the family because of its remarkable mechanism of seed dispersal. When the fruits are ripe, detachment of the stem from the cucumber body (abscission) occurs via a fracture, regulated by ethylene (23), that has been reported as one of the most rapid motions in the plant kingdom, close to the physical limit of plant movement (10, 11). Following abscission, the ripe fruit rapidly ejects both the fluid and seeds contained within its shell in a unidirectional stream—see image montage in Fig. 1*D*—within a time period of approximately 30 ms. Remarkably, the ballistic seeds attain speeds around 20 m/s and reach distances of ∼10 m from the plant.

*Ecballium* was described by the Roman naturalist Pliny the Elder (24) and, at a basic level, the dispersal mechanism is straightforward and well described by its common name—the highly pressurized fruit detaches from the stem and the seeds become ballistic as they squirt out of the fruit. The liquid jet that carries the seeds is driven by a pressure difference between the fruit interior and the external (atmospheric) pressure. That the liquid jet was driven by internal pressure was known in the 19th Century (25–27), but it was not until the 1940s that attempts were made to estimate this pressure. Obaton (28) attributed dispersal properties to the conical opening of the fruit and constructed an apparatus capable of forcing water, under known pressure, through the natural opening of a recently discharged but eviscerated specimen and inferred, by comparing droplet with seed velocities, an internal pressure of P=0.72 bar. Shortly thereafter, Lewes (29) estimated an internal pressure of 0.22 bar by inserting a syringe needle connected to a mercury manometer into a fruit, but conceded that the measurements were an underestimate due to apparatus constraints and the use of unripe specimens. He also hypothesized that fruit pressure increases and collapses internal compartments until squirting. The effect of water stress has been analyzed (30) and, more recently, this seed dispersal strategy has inspired the development of microcapsules that eject nanoparticles for on-demand drug delivery (31, 32).

However, despite its apparent simplicity, the details of the dispersal mechanism of *Ecballium*, which are key to its reproductive success, are poorly understood and a number of outstanding questions remain. Why does the plant change its shape prior to ejection? (Fig. 1*C*.) How is elasto-hydraulic energy in the pressurized fruit converted to kinetic energy? What is the velocity distribution imparted to the ballistic seeds? How do these different mechanisms influence the spatial distribution of seed landings?

To answer these questions, we combine experiments, high-speed videography, quantitative image analysis, and mathematical modeling. In doing so, we are able to quantify the transfer of energy responsible for seed projection. Through this systematic process of collecting data at different spatial and temporal scales, we uncover several previously unreported elements to the dispersal mechanism, which we hypothesize contribute to the overall effectiveness of seed dispersal. We test this hypothesis by simulating the full dispersal process—pressure build-up, fruit detachment, seed launch, and ballistics. By iterating over several plant generations, we are then able to quantify the spread of new plants starting from a single original, thus providing a measure of reproductive success and sensitivity to key parameters on a population scale. In particular, we show how the coordination of mechanical events prior to and during seed ejection contributes to dispersal success in *Ecballium*. We also investigate the dispersal of several hypothetical mutant plants, in which the development proceeds differently; this comparative analysis highlights how well-suited *Ecballium* is for efficient dispersal. Taken together, our analysis provides key insight into the evolutionary adaptations of this unique plant from inert to explosive fruits.

## 1. Methods

### 1.1. Experiments.

Plant material was acquired from the living collection of the Oxford Botanic Garden (grown under ambient conditions). Fruit were then removed to the laboratory, with their stems intact; the stems were placed in water and the fruit monitored either using a high-speed video camera or time-lapse photography, or subject to experimentation over the course of days prior to seed ejection. Additionally, measurements of the compass orientation of ripe cucumbers was performed on living specimens in Kew Gardens (London, UK) and in the wild (Zaragoza, Spain). Experimental techniques are summarized below, with further detail provided in *SI Appendix* as indicated.

#### 1.1.1. Imaging.

Time-lapse photography was performed on near-ripe specimens to investigate changes in the stem and fruit prior to seed ejection (a time-lapse video is available in Movie S2). A camera acquired images every 30 min for several days, continuing until the fruit ejected its seeds (N=6; data reported for fruits that remained within the optical plane during imaging). Seed ejection was instead imaged using a high-speed camera (Phantom Miro 310) at a rate in the range 3,200 to 8,600 fps (a slow motion video is also available, see Movie S3). Natural (i.e. nonforced) ejection events were captured using an image-based autotrigger (N=4). From the acquired images, we measured the initial speed of individual seeds and the time of their ejection after the onset of abscission and the fruit launch angle during seed ejection.

#### 1.1.2. Geometry, stiffness, and mass measurements.

**Fig. 2.**
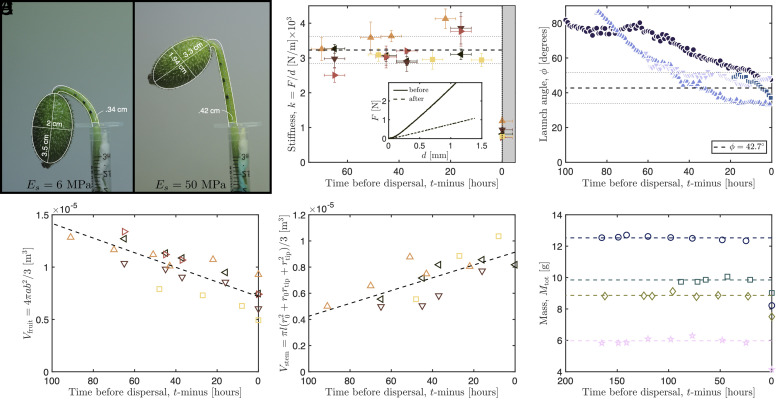
Build-up to launch. (*A*) Images of fruit reorientation 92 h prior to launch and within 30 min of launch, including model fits to stem (*SI Appendix*, section 2B) and measured fruit dimensions. (*B*) “Spring stiffness” measured from flat-punch indentation tests before and after seed ejection. Individual fruits are represented by different markers and the average pre-ejection stiffness, k=3.23±0.39 N/mm, is indicated by dashed and dotted horizontal lines, while the vertical dashed line indicates launch time. *Inset*: exemplar raw force–displacement data from indentation tests. (*C*) Launch angle ϕ (defined in Fig. 1*C*) approaches ∼40 degrees prior to launch; dashed and dotted lines indicate the mean launch angle at the onset of abscission ϕ(t=0)=42.7±8.9 degrees. (*D*) Approximate fruit volume, $V_{\mathrm{fruit}}=4\pi ab^{2}/3$ decreases prior to launch, where a and b are semimajor and semiminor axes of the fruit, respectively; the dashed line represents the best linear fit to the data. (*E*) Approximate volume of the stem, $V_{\mathrm{fruit}}=\pi l\left(r_{0}^{2}+r_{0}r_{\mathrm{tip}}+r_{\mathrm{tip}}^{2}\right)/3$, based on the stem taking the form of a conical frustum of length l and with $r_{0}$ and $r_{\mathrm{tip}}$ the radii at the base and tip of the stem, respectively. Data shown in (*B*, *D*, and *E*) acquired from the same samples, where available. (*F*) Mass of fruit plus stem section, $M_{\mathrm{tot}}$, prior to launch; dashed lines represent the average mass of each cucumber.

The shape and size of fruits, seeds, and stems were measured either using calipers or from calibrated images (see, Fig. 2*A*, for example). Flat-punch indentation tests (with an indenter of diameter 1.64 mm) were performed on cucumber fruits (N=11; data reported for ≥48 h periods prior to seed ejection) using a structural testing system (Instron 3345). Force–displacement curves (see *Inset* of Fig. 2*B*) show a linear relationship, allowing the *indentation stiffness*, defined as the gradient of force–displacement curves k=F/d, to be determined as a function of time before seed dispersal, see Fig. 2*B*. The Young’s modulus of the shell of the fruit, $E_{f}$, was measured by performing similar indentation tests on sections of dissected shell. Further details of how measurements of the stiffness were related to the internal pressure of the fruit are given in *SI Appendix*, section 1A. Additionally, fruits, with stem intact, were weighed periodically (N=6; data reported for ≥48 h periods prior to seed ejection) using an analytical balance (Mettler Toledo).

### 1.2. Mathematical modeling.

To complement the experiments described above, we developed a suite of mathematical models at different scales, describing the mechanics of several distinct components: the pressurized fruit, the stem, the rotation of the fruit following detachment, the ballistic trajectories of the seeds, and the spatial distribution of seeds over several plant generations.

These components are described further below. A full description of the models, including underlying assumptions, how they are parameterized, and how different model components are linked, is provided in *SI Appendix*, section 2.

## 2. Results

### 2.1. Seed and Fruit Characteristics.

The fruit of *Ecballium* is approximately ellipsoidal, with major and minor axes of about 4 and 2 cm respectively (Fig. 2*A*). It is attached at the end of the plant stem. Typically, a plant produces dozens of fruits which are orientated in different directions. A fruit contains ∼50 seeds of (wet) mass 25.0±2 mg (N=96) that roughly take the shape of a flattened ellipsoid of length $l_{\mathrm{se}}\approx 5$ mm, width $w_{\mathrm{se}}$≈3 mm and thickness $t_{\mathrm{se}}\approx 2$ mm. The seeds are immersed in a mucilaginous fluid matrix (which is comparable in density to water, $\rho_{\mathrm{fl}}\approx 1,000$ kg m^−3^) and occupy ≈6% of the volume within the fruit (details given in *SI Appendix*, section 1B). The shell of the fruit is h=3.20±0.24 mm (N=4) thick and measured Young’s modulus $E_{f}=0.50\pm 0.09$ M Pa. A Computed Tomography (CT) scan of an intact cucumber, see Fig. 1*B* (and visualization in *SI Appendix*), shows that the seeds are distributed in pairs in locules (chambers) along the major axis of the fruit. Individual pairs are connected to four vasculatory pillars (placentae) that are located at 90-degree intervals around the long axis of the fruit. The aperture through which the seeds pass is comparable in diameter to the width of the seeds which, on ejection, forces the seeds to align their long axis in the direction of the liquid jet.

### 2.2. Build-Up to Launch.

The stored energy in the turgid fruit is converted to kinetic energy of the jet and seeds upon ejection. Our indentation measurements in Fig. 2*B* show small variations in the indentation stiffness prior to launch, but consistently show a stiffness prior to launch that is around 3.5 times larger than that after launch, which corresponds to a pressure difference Δp≈1.7bar (see *SI Appendix* for details). The higher the pressure, the higher the velocity of the seeds (see Section 2.3 below), and thus one might expect that evolution would favor the highest possible pressure that the fruit tissue can withstand. However, the angle at which the seeds are launched is equally important in determining the horizontal distance attained from the mother plant. For example, if the fruit were to launch from a nearly vertical orientation, all seeds would fire straight up, landing very near to the mother plant. Hence, in this scenario, increasing internal pressure would not increase seed dispersal.

A delicate balance between pressure and fruit orientation was observed in a vital developmental sequence in time-lapse videos: In the build-up to launch, the inclination of fruits changes from an approximately vertical position to an angled orientation with a launch angle better suited for increased seed dispersal distances, see Figs. 1*C* and 2*C* (and the time-lapse video, Movie S2). The orientation angle of the fruit at launch was observed to be $\phi \approx 42.7\pm 8.9^{{}^{\circ}}$ relative to the horizontal (N=8; data from both time-lapse and high-speed images), which is slightly below the optimal launch angle of $45^{{}^{\circ}}$ usually expected. Accounting for drag decreases the launch optimal angle in general (3, 33); detailed calculations of the optimal launch angle with the drag relevant for *Ecballium* seeds are given in *SI Appendix*, section 2.E.1 of the SM and show that the optimal launch angle lies in the range $37^{{}^{\circ}}≲\phi ≲44^{{}^{\circ}}$. The reorientation of the fruit is a continuous process occurring over the period of several days prior to ejection. We also observed a qualitative trend in undisturbed fruits growing in the Oxford Botanic Garden: ripe fruits [detectable by a more yellow shade (28)] generally were oriented closer to 45 degrees from the vertical, whereas less ripe fruits (with a more green shade) generally were oriented closer to the vertical, see Fig. 1*A*.

The pre-ejection reorientation of the fruit may be understood in terms of observed changes to the stem during the days prior to ejection. The time-lapse videos show that the stem becomes significantly straighter and longer, increasing in radius by approximately 20%, and increasing in length by approximately 45%. The stem forms a thick-walled hollow tube, and this expansion suggests an increase in internal pressure. During the same time period, the fruit itself undergoes an active contraction, decreasing in volume; see images in Fig. 2 *A* and *D*, where we provide approximate volumetric measurements based on the ellipsoidal form of the fruit. Simultaneously, the stem increases in volume; see Fig. 2*E*, where we provide volumetric measurements assuming the stem takes the form of a conical frustum. However, in monitoring the combined mass of fruit plus stem over several days prior to launch, we detect no appreciable change; see Fig. 2*F*. This suggests that reorientation of the fruit is driven by a redistribution of fluid from the fruit, which contracts, to the stem, which expands and elongates. Accordingly, pressure in the fruit decreases prelaunch to gain a better orientation. Indeed, the geometric changes in the stem have the effect of straightening it from its initial configuration; the stiffer stem is less bent over by the weight of the fruit (Fig. 2*A*). To quantify this effect, we model the stem as an elastic rod with tapered cross-section (details given in *SI Appendix*, section 2A). When the fruit is attached to the stem, it provides a couple (a combined force and moment) to the tip of the stem. For given properties of the fruit (mass and length), the shape of the deformed stem supporting the fruit may be computed from the balances of linear and angular momenta. Stem dimensions were extracted from laboratory images, and an effective Young’s modulus $E_{s}$ was estimated both before and after fluid redistribution by fitting the deformed shape to lab images (Fig. 2*A*). An excellent fit was produced with the single fitting parameter and shows an approximately five fold increase in $E_{s}$ over the time period from 4 d prior to launch. The stiffening of a stem under increased pressure, lengthening, or tissue-tension is well known in other plants and due to the anisotropic stiffening of the fiber-reinforced outer layers of epithelial cell (34, 35). We hypothesize that a similar mechanism is at play in *Ecballium*, though investigation of such properties is beyond the current work.

### 2.3. Seed Ejection.

**Fig. 3.**
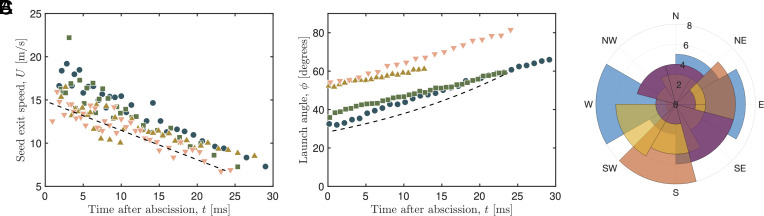
Seed ejection. (*A*) Seed exit speed measured as a function of time following abscission. (*B*) Orientation of the fruit following abscission from the stem shows that the fruit rotates at a constant rate as it falls. (*C*) Angular distribution of launch orientations, as measured in N=4 plants with a mean of n=26±6 ripe cucumbers each. In (*A*) and (*B*) markers represent experimental measurements, and the dashed lines represent model predictions, see *SI Appendix*, section 2 for details of calculations.

Upon dehiscence, the fluid and seeds stream through the fruit aperture. From images of seed ejection obtained at high frame rate, we measured the initial speed of individual seeds and the time of their ejection after the onset of abscission, see Fig. 3*A*. These data show that the speed of ejection decreases with time after abscission, and further, that this decrease is approximately linear. This observation is consistent with the intuitive picture that the high pressure within the fruit (which drives the fluid flow) is caused by the fruit containing excess liquid volume, ΔV, which decreases as liquid is expelled: As liquid is expelled, the excess volume liquid decreases, along with the driving pressure and so the speed at which fluid is ejected therefore also decreases. More quantitatively, linear elasticity theory for a shell (36, 37) shows that the driving pressure difference, Δp(t)∝ΔV(t), which can also be related to the fluid ejection speed U(t) using Bernoulli’s principle (38) so that $U\left(t\right)={\left(2\Delta p/\rho_{\mathrm{fl}}\right)}^{1/2}\propto \Delta V{\left(t\right)}^{1/2}$. Finally, we note that, by conservation of volume, the rate of change of the excess liquid volume $\dot{\Delta V}\propto U\propto {\left(\Delta V\right)}^{1/2}$ so that ${\left(\Delta V\right)}^{1/2}\propto t_{*}-t$ for some constant $t_{*}$—this immediately gives that the speed of ejection $U\left(t\right)\propto t_{*}-t$, as observed experimentally (Fig. 3*A*). (More details of this calculation, including estimates of the relevant prefactors, are given in *SI Appendix*, section 2D.)

A key step in the above calculation is the use of Bernoulli’s principle to write the excess pressure $\Delta p\left(t\right)\;=\;\frac{1}{2}\rho_{\mathrm{fl}}U{\left(t\right)}^{2}$. Such an assumption is justified by the inertial nature of the flow (the jet Reynolds number Re≈20m/s×2.5mm/10−6ms−2=5×104≫1). Nevertheless, an independent test of this assumption can be gained from comparing the initial jet-ejection speed, $U_{0}\approx 16.4$ ms^−1^, with that expected based on the measured excess pressure prior to dehiscence, Δp(0)≈1.7bar (see *SI Appendix* for details), i.e. $U_{0}^{\mathrm{pred}}\approx {\left(3.4\times 10^{5}\;\mathrm{Pa}/1,000\;\text{k}\text{g}\;{\text{m}}^{-3}\right)}^{1/2}\approx 18.5\;\text{m}\;{\text{s}}^{-1}$. These estimates of the initial velocity of ejection agree to within ≈10%, validating a key step in our modeling.

The high-speed images of the fruit immediately following dehiscence also reveal that as it falls, the fruit rotates away from the stem at a nearly constant rate (Fig. 3*B*). Moreover, this rotation is in a direction that increases the launch angle of the seeds (relative to the horizontal) and occurs on the same timescale as the ejection of the seeds—the launch angle of each seed varies with its position in the launch sequence. The direction and rate of rotation were consistent among the fruit launches we observed, suggesting that this rotation is a robust feature. A variable launch angle leads to a wider distribution of landing spot of the seeds, which likely imparts an evolutionary advantage. To understand the mechanism behind this rotation, we first note that the fruit itself is approximately symmetric about the major axis, and the aperture is also symmetric. Moreover, the seeds are distributed uniformly and symmetrically within the fruit, as can be observed from the CT scan (Fig. 1*B*). Therefore, the reaction force on the fruit due to the ejection of the jet and seeds cannot impart a consistent and robust torque on the fruit itself. Rather, we postulate that the rotation derives from the interaction between the fruit and the stem during the dehiscence event at the very start of the ejection. Once the fruit has broken free from the stem, the stem recoils away from the fruit (due to breaking free from supporting the mass of the fruit). During the first couple hundred microseconds of ejection, while the fruit and stem are breaking apart but still in contact, the tip of the stem rotates away from the fruit, and the balance of angular momentum implies that the fruit should rotate in the opposite direction. To provide an estimate of the rotation, we develop an idealized model (*SI Appendix*, section 2C) of the fruit/stem interaction during these first instants of ejection, introducing inertia to the elastic rod framework and assuming that the moment applied by the fruit to the end of the stem continuously decreases to zero over the dehiscence time scale; this analysis predicts a rotation rate that is consistent with our experimental observations (see the dashed line in Fig. 3*B*).

### 2.4. Ballistics.

The ballistic trajectories r(t) of individual seeds of mass m satisfy the kinematic relation

$$m\ddot{r}\left(t\right)=-mge_{z}-F_{d}\frac{\dot{r}\left(t\right)}{|\dot{r}\left(t\right)|},$$

where g is the gravitational acceleration, acting in the vertical direction $e_{z}$, and $F_{d}$ is the drag force (*SI Appendix*, section 2E). For given initial seed velocities and launch angles, obtained either via mathematical modeling or empirical best fits to data, we integrate numerically the trajectories, from which we compute the horizontal distance from the mother plant at which each seed lands. We first compute the landing distance for the seeds of a single fruit. Our results indicate that the seeds are projected over a horizontal distance between ∼4 and ∼12 m. This is in agreement with previous measurements of a 5 to 6 m range (28) and more recent reports of a dispersal distance up to 10.2 m, (see ref. 39 and references therein).

## 3. Comparative Analysis

Our analysis has uncovered several key ingredients contributing to the dispersal of *Ecballium* seeds. To summarize:

I On the timescale of weeks prior to dispersal, the fruit becomes highly pressurized by a mucilaginous fluid.
II In the days before dispersal, some of the fluid is redistributed from fruit to stem, thereby stiffening the stem and rotating the fruit away from the vertical.
III In the first hundreds of microseconds of ejection, the stem detaches from the fruit, recoiling away and imparting a counterrotation to the fruit.
IV The seeds are ejected with an exit speed and launch angle that depend on their sequence: The exit speed decreases (because of capsule depressurization) while the launch angle increases (because of the fruit’s rotation).

We are able to simulate the distribution of seeds dispersed from a single plant by linking the mathematical models of the pressurized fruit, stem stiffening, fruit rotation, and seed ballistics in a way that incorporates key physical/material parameters and developmental events. The distribution is highly dependent on the specifics of the above ingredients, and it is natural to ask how well suited the fruit of *Ecballium* is for dispersal. To investigate this, we first extend from the simulation of a single fruit to the simulation of all the fruits of a given mother plant. Measurements of the launch azimuth of ripe cucumbers, see Fig. 3*C*, show that cucumbers are distributed relatively uniformly around the compass orientations. We thus simulate the trajectories of seeds ejected from 30 different fruits (30 seeds per fruit) with initial velocity vector computed from our mathematical model (*SI Appendix*, section 2E), such that the compass orientation, when viewed from above, is drawn from a uniform random distribution. Since offspring require some minimum distance between neighbors for survival, the number of next-generation plants depends sensitively on the spatial distribution of dispersed seeds. To extend our simulation to the next generation, we assume a given survival rate (probability that a seed produces a new plant) and a minimum distance between nearest offspring (for details, see *SI Appendix*, section 2F, which includes the results of simulations under different choices for the survival probability and minimum distance). We can then simulate seeds ejected from each second-generation plant, leading to third-generation plants, and so on. In this way, we can build up a picture of the distribution of plants over multiple generations starting from a single original plant, and investigate how the spreading is impacted by a change in parameters and/or development.

**Fig. 4.**
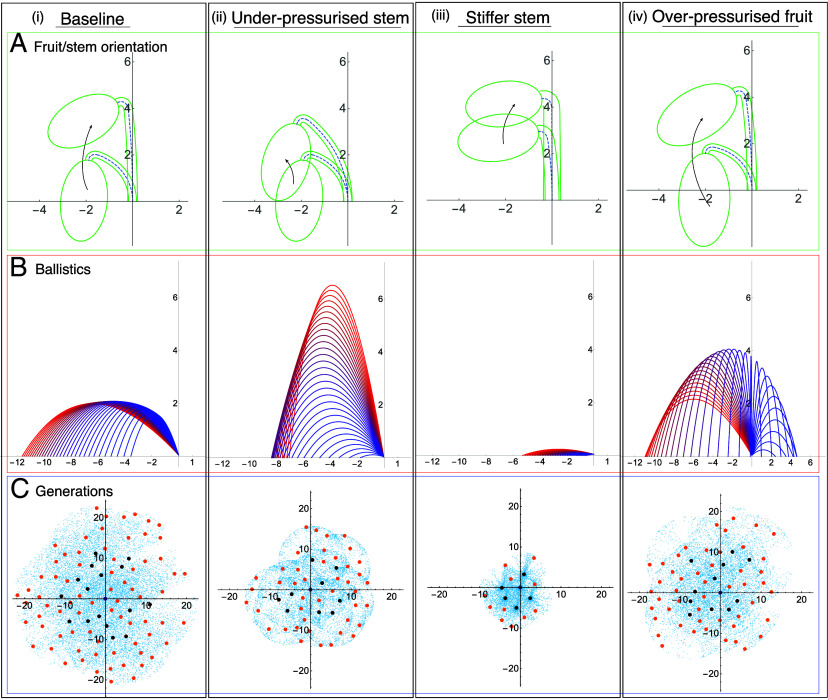
Comparative analysis. (*A*) The shape of fruit and stem, measured in cm, before and after reorientation due to fluid redistribution from fruit to stem. (*B*) Resulting ballistic trajectories for N=30 ejected seeds with initial velocities and launch angles computed from fluid jet and fruit rotation models; units in m. (*C*) Simulation of the spatial distribution of seeds over 3 generations (the central purple dot shows initial plant location, black dots are second-generation plants, blue dots show seed distribution at second-generation seed dispersal, and orange dots show locations of third-generation plants); units in m. Results are shown for parameters describing (*i*) the conditions observed in the laboratory (Fig. 2), (*ii*) a weak or underpressurized stem, (*iii*) a stiffer or overpressurized stem, and (*iv*) an overpressurized fruit.

The results of our comparative analysis are shown in Fig. 4 (for details, see *SI Appendix*, section 2G). The base case appears in the *Left* column, Fig. 4*A*–*C*, *i*. Here, we have simulated dispersal with parameters corresponding to those that we have extracted from laboratory observations on real *Ecballium* (all parameters given in *SI Appendix*), i.e. this case models dispersal seen in nature. The remaining columns consider particular developmental and/or parametric variations. For each case, we show the orientation of fruit/stem before and after reorientation (*Top* row), the trajectories of seeds for a single fruit (*Middle* row), and an aerial view of the distribution of seeds over the course of three generations (*Bottom* row).

In Fig. 4*A*–*C*, *ii*, we simulate an underpressurized stem, i.e. a plant for which a smaller degree of fluid redistribution occurs. Here, the fruit is more pressurized at the point of seed ejection, giving higher seed velocities, but the launch angle is nearly vertical. Thus, the seeds are ejected high in the air but do not achieve significant horizontal distance; accordingly very few seeds survive to subsequent generations.

Fig. 4*A*–*C*, *iii* simulates a thicker, and therefore stiffer stem, that deforms less under the weight of the fruit. With all other parameters and developmental events the same, in this case the increased stiffness and redistribution of fluid results in a nearly horizontal launch angle. The stiffer stem therefore undergoes less recoil and the fruit rotates less as seeds are launched. The consequence is a narrower seed distribution and with comparatively low horizontal distance from the plant, leading to few seeds surviving at the third generation.

The final case we consider is an overpressurized fruit obtained by a slight increase in volume (Fig. 4*A*–*C*, *iv*). Here, the increased force on the stem (due to the heavier fruit) creates a larger recoil of the stem following detachment and thus leads to a higher fruit rotation. This creates a much higher variance in launch angle, as can be seen by examining the trajectories in this case, Fig. 4*B*, *iv*. Despite the high variance of seed distribution from a single fruit, this is not a cost-effective strategy, as some seeds fire almost straight down, while others are launched backward. Thus, the number of plants surviving at the third generation is again still lower than the base case.

Fig. 4 highlights how changes to the launch angle (via stem stiffness), degree of fruit pressurization, and/or fruit counterrotation (via degree of stem recoil) can all have a strong impact on the dispersal. The question remains to what degree *Ecballium* has been bioengineered by evolution to achieve successful dispersal, i.e. are the parameters we have measured optimal in some sense, and how fine-tuned must they be? As a partial answer to this, we focus on the key developmental event of fluid redistribution from fruit to stem in the days prior to launch, and ask how the dispersal would vary if more or less redistribution occurred. To this end, we define the relative fluid redistribution parameter, β, which characterizes the degree of fruit deflation and stem inflation due to fluid redistribution and is defined relative to the baseline values we have extracted from real specimens (defined precisely in *SI Appendix*, section 2H). A value of β>1 corresponds to more fluid redistribution than we observed, which creates a stiffer stem and less-pressurized fruit, while β<1 gives less redistribution, creating a weaker stem but more pressurized fruit. For a given β, we simulate the seed dispersal from a single fruit, and compute 7 dispersal metrics—mean seed distance, maximum seed velocity, minimum and maximum seed distances, fruit launch angle, fruit rotation, and the standard deviation of seed distribution. These metrics are plotted as a function of β in *SI Appendix*, section 2H, and each shows strong and generally nonlinear dependence on β. For very small β, the launch angle is close to vertical and the fruit rotation during launch is low, so seeds fire mostly vertically, reaching a low maximum distance with low standard variation. Thus, seeds cluster at an intermediate distance. For β∼0.5, the fruit is more highly pressurized than the base case, creating a larger maximum velocity, but the weak stem and more massive fruit creates an increased fruit rotation, resulting in many seeds landing close to the plant. For β∼2, the less pressurized fruit generates lower velocities, and the stiffer stem results in a more horizontal launch angle and less fruit rotation, leading to a lower mean seed distance compared to the base case β=1.

**Fig. 5.**
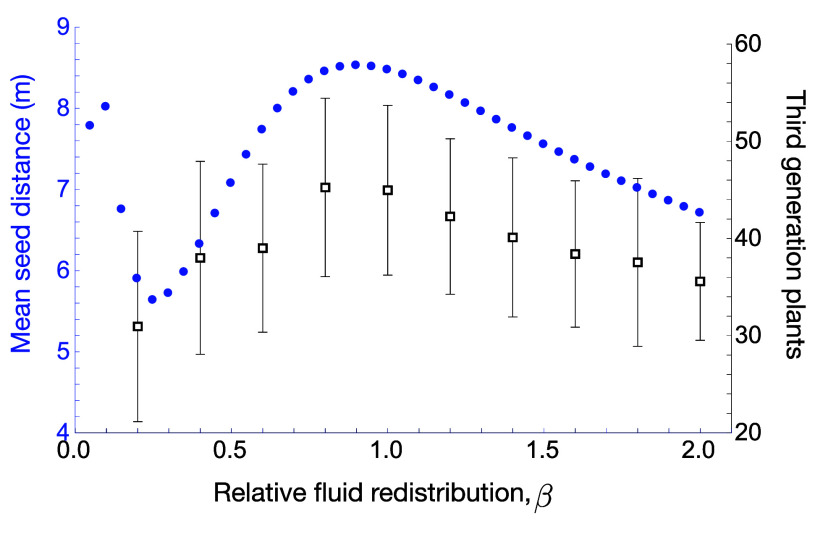
Impact of fluid redistribution. Ballistic seed dispersal was simulated for varying degrees of fluid redistribution from fruit to stem, denoted by the parameter β, such that β=1.0 corresponds to the degree we have extracted in laboratory measurements. Here, we plot the mean seed distance of seeds dispersed from a single fruit (blue dots, *Left* axis). We also display predictions from the generational model. We performed 50 generational simulations at each value of β, computing the number of third-generation plants; we plot the average (black squares, *Right* axis), with errors bars indicating the SDs.

To examine how these characteristics may combine to determine overall reproductive success, we also computed the number of third-generation plants predicted from the probabilistic model, averaged over N=50 simulations. The mean seed distance and number of third-generation plants are plotted together in Fig. 5; interestingly, each displays an internal maximum near β=1. Our analysis demonstrates precisely why too little or too much fluid redistribution is detrimental to dispersal, while the slightly broad peak of the curves in Fig. 5 implies that dispersal success may not require perfect fine-tuning but may be attainable over a small range of parameters. Our results also suggest that mean seed distance from a single fruit may serve as a good proxy for predicting dispersal success for a plant over several generations; of the metrics considered, mean distance most closely followed the quantitative shape of the predicted number of third-generation plants.

## 4. Discussion

Seed dispersal is central to plant population dynamics and the survival of a species. A diversity of seed dispersal mechanisms has evolved across the plant kingdom, and accordingly a vast scientific literature exists aiming to understand both the mechanisms and effectiveness of dispersal strategies (40). Uncovering physical mechanisms of seed dispersal is a crucial component in predicting ecological dynamics e.g., the potential for invasive migratory species in a given ecology; in understanding the effects of environmental changes due e.g., to climate change; and, in some cases, in inspiring technologies. Here, we have uncovered and quantified key mechanical features underpinning one of the most remarkable dispersal mechanisms in the plant kingdom, the explosive dispersal of the squirting cucumber, *E. elaterium*. In particular, the key mechanical features behind the success of Ecballium’s seed dispersal mechanism involve i) the reorientation of fruit prior to launch such that the launch angle is close to optimal for a projectile (a process that we infer to be driven by fluid redistribution from the contracting fruit to the expanding stem) and ii) a combination of a decrease in seed launch speed with a counterrotation of the fruit during ejection, which together ensure broad dispersal of the seeds.

*Ecballium* is a monotypic genus, containing only one species, *E. elaterium*. The most closely related genus is *Bryonia* which contains 12 species found mainly in dry areas of Turkey, Syria, Iran, Iraq, Afghanistan, and Pakistan, with four adapted to deserts. These two genera diverged from their sister genus—*Austrobryonia*—which reached Australia 36 (50 − 24) Mya; *Austrobryonia* also occurs in arid environments (41). Both *Bryonia* and *Austrobryonia* contain species with a climbing habit and tendrils (although some are prostrate), and produce berries. The berries of *Bryonia* are small, and bird- and water-dispersed (42); those of *Austrobryonia* are larger and consumed whole by terrestrial birds such as emus (*Dromaius novaehollandiae*) and bustards (*Ardeotis australis*).

Birds or ocean currents may have played a role in long-distance dispersal and range expansion of the ancestral lineage. While the ejection of copious fluid in *Ecballium* might seem energetically expensive or even wasteful in a water-constrained environment, most species within the *Ecballium*-*Bryonia*-*Austrobryonia* clade are adapted to mesic-to-arid environments, and produce fleshy berries. This suggests the production of mucilage-rich fruits under water stress was ancestral and preadapted explosive dispersal. In other words, the shift to ballistic dispersal in *Ecballium* may not have incurred an additional water cost, or any cost incurred could have been offset by a selective advantage associated with reduced water competition.

The paucity in living ancestors and fossil relatives of *Ecballium* makes it challenging to reconstruct an evolutionary pathway from inert to explosive fruits. However, a suite of anatomical, and chemical features appear to have acted as possible preadaptations, such as a high concentration of glucosides, coupled with a small pericarp aperture; a consequent build-up in osmotic pressure causing the fluid in cells around the seeds to exceed that of the force holding together the cells of the stalk abscission layer could have led to pericarp detachment. Further selective advantages may have been associated with reduced competition from the parent plant, driving an increase in ejection force and seed dispersal distance. We also speculate that concurrently dispersing seeds with their own initial water supply benefits the probability of propagule establishment yet acknowledge testing this hypothesis is beyond the scope of this report.

Our mechanical analysis demonstrates *E. elaterium* disperses seeds over a distance of about 250 times the length of the fruit from the parent plant. As well as providing a means of colonizing new environments, seed dispersal can also reduce competition among neighboring plants (43). Conspecific neighbors often have a greater detrimental effect on plant performance (survival, growth, and reproduction) than do heterospecific neighbors, a phenomenon known as negative density dependence. It can be a strong stabilizing force for population regulation, decreasing the spatial aggregation (crowding) of offspring by spreading seeds over a large area (44, 45). Perennial species tend to have more aggregated distributions than annual species (46), and the squirting cucumber in particular shows marked spatial and temporal clustering (39). Limited data exist for *Ecballium* population dynamics in vivo, and none from natural environments. A three-year study aimed at controlling the plant where it is a weed in orchards indicates clustering (39), at least in the short term. Our data suggest that ballistic autochory could enable a regular spatial-temporal pattern of dispersal that acts to reduce crowding over generations in the long term, possibly as a consequence of selection by negative density dependence.

Finally, we highlight that the redistribution of fluid from fruit back to stem and corresponding stiffening of the stem prior to seed ejection appears to be a mechanism unique to this species. The cellular/ subcellular mechanisms driving this change offer intriguing avenues for future investigations. In particular, redistribution of fluid at fast and slow time scales manifests differently in the mechanics: Rapid fluid movement during launch gives a decreasing fruit pressure (and seed launch speed), while slower fluid movement prior to launch appears to have little effect on the fruit stiffness (Fig. 2*B*), but a large effect on stem stiffness. And indeed, our comparative analysis has demonstrated that these mechanical details are critical to the successful dispersal of seeds and propagation of the plant to subsequent generations. While our approach was to extract the increased stem stiffness as a fitting parameter within an elastic rods framework, a first principles understanding of the fluid redistribution process would provide an improved understanding, and could also enable predictions of the impact of changing environmental conditions on the mechanism and ultimately on reproductive success. Other potential model improvements include a full dynamic resolution of the separation of fruit from stem and a more detailed investigation of the flow field of the fluid and immersed seeds as they are ejected. Naturally, the ability of any given specimen to produce offspring will be highly dependent on a number of environmental conditions (wind, soil, water levels, etc), which may impact the system at different levels; thus another important direction for future modeling is a general exploration of the impact of the environment on the dispersal mechanism and ultimately on reproduction, for which our framework provides a natural starting point.

## Supplementary Material

Appendix 01 (PDF)

Movie S1. CT Scan of fruit, providing detailed view of internal structure, including seed placement, size, and shell thickness.

Movie S2. Timelapse of the final days of development of a ripe fruit. Near ripe fruits were extracted from the Oxford Botanic Garden, stem intact, and placed in a supporting tube with water. Images were acquired every 30 minutes, continuing until the fruit ejected its seeds.

Movie S3. seed ejection filmed at 8600 frames per second. A nearly ripe fruit was extracted from the Oxford Botanic Garden, stem intact, and placed in a supporting tube with water. A high-speed camera remained aimed at the fruit for several days, with an image-based auto-trigger set up, so that seed ejection could be captured when it occurred naturally.

## Data Availability

Experimental data sets and the *Mathematica* notebooks reproducing model output are available in the public depository (47).
