# Supplementary material for: Uncovering the mechanical secrets of the squirting cucumber
Source: Proc Natl Acad Sci U S A. 2024 Nov 25;121(50):e2410420121. doi: 10.1073/pnas.2410420121 (PMC11648854; doi:10.1073/pnas.2410420121)
Supplement: Supplementary file 1 — Appendix 01 (PDF) [file pnas.2410420121.sapp.pdf]

Supplementary Information for

Uncovering the mechanical secrets of the squirting cucumber

F. Box, D.E. Moulton, D. Vella, Y. Bhagotra, T. Lowe, A. Goriely, & C. Thorogood

This PDF file includes:

Figs. S1 to S7
Tables S1 to S2
SI References

Contents

1 Experimental details **2**
- A Measurement of internal pressure . . . 2
  - A.1 Measurement of the force–indentation law . . . 2
  - A.2 Models of pressurized fruit . . . 2
- B Visualizing the cucumber seeds . . . 4

2 Mathematical models **4**
- A Stem model . . . 4
- B Approximating bending stiffness . . . 7
- C Fruit rotation . . . 8
- D Fluid/seed ejection . . . 11
- E Ballistic trajectories . . . 12
  - E.1 Optimal launch angle . . . 13
  - E.2 Trajectories of multiple seeds . . . 13
- F Plant generations . . . 14
- G Comparative analysis parameter changes . . . 15
- H Fluid redistribution . . . 15

3 SI video legends **17**

1. Experimental details

A. Measurement of internal pressure. To estimate the internal pressurization of the fruit in the build-up to launch, we used indentation of the fruit at its side. Here we first describe how this indentation experiment was performed to determine the linear indentation stiffness,

$$k = \lim_{\delta \to 0} \frac{F}{\delta}. \qquad [S1]$$

We then discuss the mathematical models of the indentation of pressurized ellipsoids that were used to infer the internal pressure.

A.1. Measurement of the force–indentation law. The fruit's surface is approximately that of a prolate spheroid; in Cartesian coordinates centered on the center of the fruit we may then write

$$(x/a)^2 + (y/b)^2 + (z/c)^2 = 1,$$

where the semi-axes are of length $b = c \approx a/2$. Indentation tests were performed at $z = c\,(= b)$.

We performed indentation experiments over the course of days prior to seed ejection, using a flat-punch indenter of diameter 1.64 mm) connected to an Instron 3345 structural testing system to acquire measurements of the applied force as a function of indentation depth. The gradient of force-displacement curves $k = F/d$ was determined from linear fits to the post-contact data for indentation depths < 1 mm; reported values represent the mean of at least three measurements.

A.2. Models of pressurized fruit. To infer the internal pressure from the measured value of k, we model the fruit as an ellipsoidal shell with internal pressure p, shell thickness h and shell modulus E_f. The problem of the point indentation of such a shell has been considered previously (1, 2).

We use the results of Sun & Paulose (2), who related the small indentation stiffness k, defined in Eq. (S1), to the mechanical properties of the shell (its bending stiffness B and stretching stiffness Y), its geometry

(the radii of curvature $R_x = a^2/b$ and $R_y = b$ local to the indenter) and the internal pressure p. In particular, they showed that

$$k(p) = 8\pi^2 \frac{B\tau}{\ell_b^2} \left[\int_0^{2\pi} (1+\beta \sin^2\theta)^{-1} \frac{\tanh^{-1}(1-\Upsilon^2)^{1/2}}{(1-\Upsilon^2)^{1/2}} \, \mathrm{d}\theta \right]^{-1}, \quad \text{[S2]}$$

where

$$\Upsilon = \tau^{-1} \frac{1-\beta \sin^2\theta}{1+\beta\sin^2\theta}, \quad \text{[S3]}$$

$$\beta = 1 - R_y/R_x = 1 - b^2/a^2, \quad \text{[S4]}$$

is a geometrical parameter,

$$\ell_b = \left(\frac{BR_y^2}{Y} \right)^{1/4} \quad \text{[S5]}$$

is the characteristic horizontal length scale of shell deformations in the absence of pressure, and

$$\tau = \frac{pR_y^2}{4(BY)^{1/2}} \quad \text{[S6]}$$

is a dimensionless measure of the pressure within the shell.

While the integral in Eq. (S2) is not amenable to analytical evaluation, it may readily be computed numerically for given values of the parameters β and τ. More difficult is the fact that k depends on the (unknown) bending and stretching moduli, B and Y. However, it is readily shown that the unpressurized stiffness, $k_0 = k(p=0)$ is given by

$$k_0 = 8\sqrt{1-\beta}(BY)^{1/2}/R_y. \quad \text{[S7]}$$

The ratio $k(p)/k_0$ can therefore be written as

$$\frac{k(p)}{k_0} = \frac{\pi^2}{\sqrt{1-\beta}} \tau \left[\int_0^{2\pi} (1+\beta \sin^2\theta)^{-1} \frac{\tanh^{-1}(1-\Upsilon^2)^{1/2}}{(1-\Upsilon^2)^{1/2}} \, \mathrm{d}\theta \right]^{-1}, \quad \text{[S8]}$$

which is therefore a function of β, R_y, and τ.

Equation Eq. (S8) shows that the ratio $k(p)/k_0$ is a function of τ (which is unknown) and β (which is readily estimated). (In our experiments $0.65 \leq \beta \leq 0.78$, with mean 0.73.) Figure S1 shows that with this range of β, the dependence of τ on $k(p)/k_0$ is only slightly sensitive to the value of β.

The numerical results presented in fig. S1 can be used to estimate the value of τ immediately before seed launch in each of the experiments presented in fig. 2B of the main text. The experimentally measured ratio $3.1 \lesssim k(p)/k_0 \lesssim 4.2$, which corresponds to $1.4 \lesssim \tau \lesssim 2.4$. Since τ can itself be rewritten

$$\tau = 2\sqrt{1-\beta} \frac{pR_y}{k_0}, \quad \text{[S9]}$$

we readily calculate the corresponding internal pressure, which we find to be $p \approx 1.70 \pm 0.15$ bar.

The pressure $p \approx 1.70$ bar estimated using indentation is significantly larger than the reported value of 0.72 bar (3) that was inferred from analogue experimentation with eviscerated fruits. However, we note that, in combination with the model based on Bernoulli's principle presented in §2D, this corresponds to an initial jet speed $U = (2p/\rho_{\mathrm{fl}})^{1/2} \approx 18.4$ m/s, which is in reasonable agreement with our own experiments.

It will also be useful later to have estimates for the typical stretching modulus of the shell, $Y = E_f h$. We note here that, under the assumption that $B = Eh^3/[12(1-\nu^2)]$, this can be inferred from the measured value of $k(p=0)$ as

$$Y = \frac{4}{\sqrt{3(1-\nu^2)}} \frac{(R_x R_y)^{1/2}}{h} k_0 = \frac{4}{\sqrt{3(1-\nu^2)}} \frac{a}{h} k_0. \quad \text{[S10]}$$

With the measured value $h = 3.2$ mm, we find that $Y \approx 2 \pm 0.6$ kN m^{-1}, which corresponds to a Young's modulus $E_f \approx 640 \pm 200$ kPa, which is in good agreement with the measured value of 0.50 ± 0.09 MPa acquired from indentation tests on fruits shells.

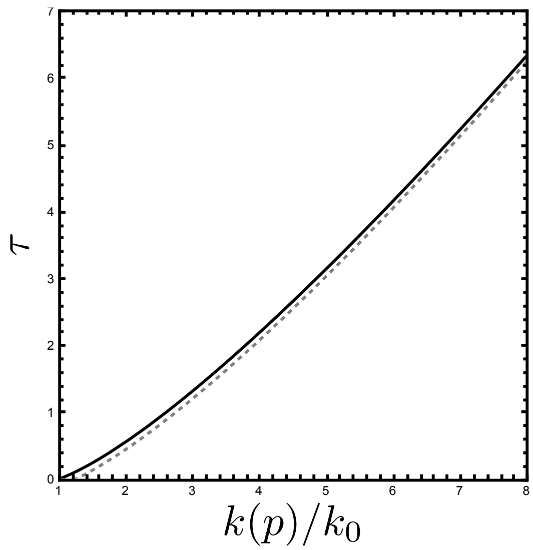

Fig. S1. Evaluation of the dimensionless pressure from the experimentally measured values of $k(p)/k_0$. Here, the numerically determined behavior of Eq. (S8) is used to plot the effective τ from the measured $k(p)/k_0$; results are shown for $\beta = 0.646$ (dashed grey curve) and $\beta = 0.783$ (solid black curve).

B. Visualizing the cucumber seeds. A ripe specimen was scanned at the University of Manchester using the Henry Moseley X-ray Imaging Facility's custom Nikon 225 kV X-ray tomography system. The system is equipped with a static tungsten reflection target source, a PerkinElmer 4096 x 4096 pixels 16-bit amorphous silicon flat-panel detector, and has a max power of 450 W. The specimen was wrapped in bubble wrap and held in place during the scan. X-rays were generated using a voltage of 120 kV and a current of 183 μA. The screen to detector distance was 1138.60 mm and the screen to object distance 136.56 mm; each radiograph (projection) was acquired using binning of two, giving an effective detector size of 2024×2024 pixels, with an effective pixel pitch of 100 μm. The image resolution was $1458 \times 1388 \times 2024$ voxels of size 23.99 microns. The total number of projections was 2001. The 3-D volumes were reconstructed from the projection data using Nikon's CT Pro 3-D software (Nikon X-Tek Systems Ltd, UK). Image segmentation was performed using machine-learning (ML) assisted smart segmentation techniques in IPSDK Explorer (Reactiv'IP, France), see Fig. S2. This involved (i) cropping the projections, (ii) using ML to segment seed outlines, (iii) dilating and eroding seed boundaries, removing image noise (small objects) from seed boundaries and filling holes within seed boundaries, (iv) masking seeds and using ML to segment the air gap that surrounds the seeds, (v) masking the seed + air gap regions and using ML to segment the cucumber shell and vasculature network, and (vi) masking the shell to determine the combined volume of the soft interior (mucilaginous fluid matrix + seeds + vasculature network). Segmented projections were rendered into visualizations using Dragonfly (Comet Technologies Canada Inc, Canada), see Fig. 1B.

From the processed CT scan we could identify 54 seeds of volume $7.23 \pm 0.32\,\mathrm{mm}^3$, with a ratio of max-to-min Feret diameter of 2.42 ± 0.07, that are each surrounded by a small air gap, and one empty air sack. The volume of the shell interior could be estimated by counting voxels, as could the total volume of seeds, which enabled estimation of the volume occupied by seeds within the fluid matrix inside the shell, found to be 5.8%.

2. Mathematical models

A. Stem model. We model the stem as a planar, inextensible, unshearable, elastic rod, with initial length L. The appropriateness of an elastic rod treatment is given by the significant aspect ratio between stem length (on the order of 5 cm) and stem radius (on the order of 0.2 cm), while the assumption of inextensibility and unshearability are standard for biological filaments. The centerline is described by the curve

$$\mathbf{r}(S) = x(S)\mathbf{e}_x + z(S)\mathbf{e}_z, \qquad \text{[S11]}$$

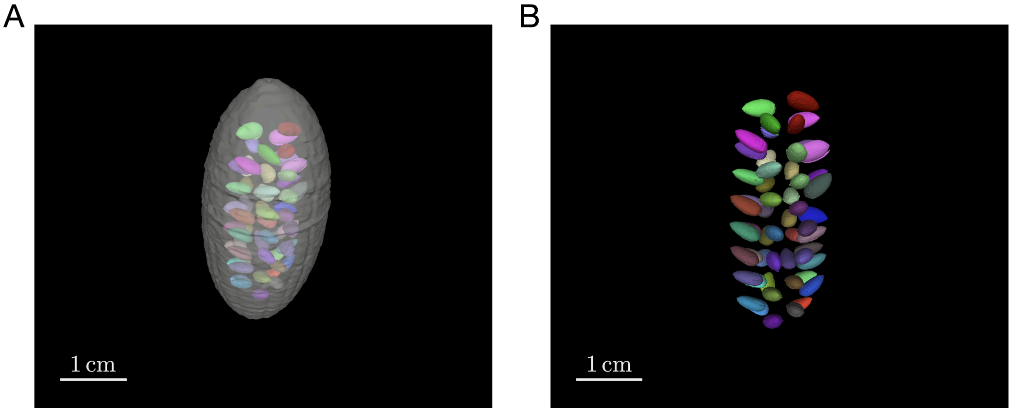

Fig. S2. **Imaging inside the shell.** A. A reconstructed CT scan of the cucumber interior shows that the seeds (coloured) are suspended inside a fluid matrix (gray). The relative volume of seeds to fluid was measured by counting the number of voxels of the segmented regions. B. The number of seeds inside the scanned cucumber, along with measurements of their volume and maximum and minimum Feret diameter, were extracted from calibrated images using IPSDK. These measurements of seed size and shape complemented data from other fruits that was manually acquired post-ejection using calipers.

where S is the arc length parameter prior to any elongation. Here, the stem is in the (x, z) plane, such that gravity points in the negative z-direction. During development prior to seed ejection, the stem undergoes very slow changes (on the order of hours to days) in length and cross-sectional thickness. Let γ denote the factor by which the length increases[*]; in particular, if the stem extends to length l, then $\gamma = l/L$, and the arc length s in the grown configuration relates to the arc length in the initial configuration by $s = \gamma S$ (4, 5). Defining $\theta(S)$ as the angle between the tangent vector $\mathbf{r}'(S)$ and the vector $\mathbf{e}_x$, we may write

$$x'(S) = \gamma \cos\theta, \quad z'(S) = \gamma \sin\theta. \qquad \text{[S12]}$$

Due to the slow changes in material dimensions with respect to the elastic timescale, the rod may be taken to be in quasi-static mechanical equilibrium (up to the time of separation from the fruit). The shape is thus governed by the static balances of linear and angular momentum, which read (5)

$$\mathbf{n}'(S) + \mathbf{f} = \mathbf{0}, \qquad \text{[S13]}$$

$$\mathbf{m}'(S) + \mathbf{r}'(S) \times \mathbf{n}(S) = \mathbf{0}. \qquad \text{[S14]}$$

Here $\mathbf{n}(S)$ and $\mathbf{m}(S)$ respectively denote the resultant force and moment in the rod, while $\mathbf{f}$ is the total body force. Assuming that the only body force is due to self-weight, we have $\mathbf{f} = -\rho_s g \mathbf{e}_z$, where ρ is the linear density of the stem, and g is the gravitational acceleration. Since the rod remains in the (x, z) plane, the moment only has a component in the $\mathbf{e}_y$ direction, which is related to the curvature $u(S)$ by the constitutive relation

$$\mathbf{m} = E_s I_s (u(S) - \hat{u}(S))\mathbf{e}_y. \qquad \text{[S15]}$$

Here E_s is the Young's modulus for the stem, I_s is the 2nd moment of area, and $\hat{u}$ is the intrinsic curvature, describing the shape of the stem in its unloaded configuration. The curvature u is equal to the (negative of the) derivative with respect to arc length of the angle θ, i.e. $u = -\gamma^{-1}\theta'(S)$, where the factor of γ^{-1} converts to an arc length derivative[†]. Images of the stem with the fruit detached suggest that the stem

[*] In principle we could have $\gamma = \gamma(S)$, but lacking a means of quantifying any non-uniformity in the extension, for simplicity we take γ to be a constant.

[†] The negative appears due to placing the rod in the (x, z) plane, and may be derived from the relation $\mathbf{d}_3'(s) = \mathbf{u} \times \mathbf{d}_3$, where $\mathbf{d}_3 = \cos\theta \mathbf{e}_x + \sin\theta \mathbf{e}_z$ is the tangent vector and $\mathbf{u} = u_2 \mathbf{e}_y$ is the curvature vector (4)

tends to be naturally straight in its reference configuration, except for a small region near the tip that shows significant curvature and acts as an hinge. In that region bending is localized under the weight of the fruit. We account for this profile with the following form:

$$\hat{u}(S) = -C \exp\left(\frac{-\gamma(S-(L-dS))^2}{\sigma^2}\right), \qquad \text{[S16]}$$

where dS is the distance from the tip to the localized bending region, C characterizes the degree of bending, and σ characterizes the width of the hinge region.[‡] The stem is a thick-walled hollow tube with approximately circular cross-section, for which the moment of area I_s is given by

$$I_s = \frac{\pi}{4}\left(r_2^4 - r_1^4\right), \qquad \text{[S17]}$$

where r_1 and r_2 are the inner and outer radii. These values change during development, as fluid redistribution causes the stem radius to expand. However, we do not have access to measure the inner radius prior to launch. For stems with fruit detached, we find that $r_1 \approx 0.5 r_2$. We thus set $r_1 \equiv r_2/2$, which gives

$$I_s = \frac{15\pi}{64} r_2^4. \qquad \text{[S18]}$$

The radius r_2 is also a function of S due to tapering of the stem from base to tip, which is well-approximated by a linearly decreasing function of S:

$$r_2 = r_0 - m_{taper} S. \qquad \text{[S19]}$$

The values r_0 and m_{taper} can be measured from lab images, with typical values $a \approx 0.2$cm, $b \approx 0.01$. Since the stem is only slightly tapered, for simplicity we approximate the density ρ_s as being constant, equal to the total mass of the stem divided by the current length γL.

While the fruit is still attached to the stem, the weight of the fruit deforms the stem, by applying both a force and moment to the tip of the stem. The fruit may be modeled as a point mass, with mass M_f located at its center of mass, which leads to the boundary conditions:

$$\mathbf{n}(L) = -M_f g \mathbf{e}_z, \qquad \text{[S20]}$$

$$\mathbf{m}(L) = a(\cos\theta(L)\mathbf{e}_x + \sin\theta(L)\mathbf{e}_z) \times (-M_f g \mathbf{e}_z) = a M_f g \cos\theta(L)\mathbf{e}_y. \qquad \text{[S21]}$$

The first equation describes the load applied by the fruit at the boundary of the stem. Since the center of mass of the fruit is not at the tip of the stem, the fruit also applies a torque to the stem, described by the second equation, noting that $a(\cos\theta(L)\mathbf{e}_x + \sin\theta(L)\mathbf{e}_z)$ is a vector pointing from the tip of the stem to the center of mass of the fruit (a is the major semi-axis of the ellipsoidal fruit). At the base of the stem, we apply clamped boundary conditions at a point that is taken without loss of generality to be the origin:

$$x(0) = z(0) = 0, \quad \theta(0) = \theta_0, \qquad \text{[S22]}$$

which completes the system. The resultant force $\mathbf{n}$ only has a component in the z direction, which may be determined explicitly:

$$\mathbf{n} = (\rho_s g(S-L) - Mg)\mathbf{e}_z. \qquad \text{[S23]}$$

Inserting this into the moment balance (S14), and using the constitutive relation (S15), we obtain a single second order equation for the angle $\theta(S)$:

$$\frac{d}{dS}\left[E_s I_s(s)\left(\gamma^{-1}\theta'(S) - C\exp\left(\frac{-\gamma(S-(L-dS))^2}{\sigma^2}\right)\right)\right] = -\gamma\cos\theta\left(\rho_s g(S-L) - M_f g\right), \qquad \text{[S24]}$$

[‡] The factor of γ appears so that the hinge region stays at the same proportional location under extension of the stem.

along with boundary conditions

$$\theta(0) = \theta_0, \quad E_s I_s(L)\left(\gamma^{-1}\theta'(L) - C\exp\left(\frac{-\gamma dS^2}{\sigma^2}\right)\right) = -aM_f g\cos\theta(L). \qquad \text{[S25]}$$

For given parameters – stem parameters $\{E_s, r_0, m_{taper}, L, \gamma, C, dS, \sigma\}$; fruit parameters $\{M_f, a\}$; and base angle θ_0 – the shape of the stem is determined as the solution of the boundary value problem outlined above. Once $\theta(S)$ is known, the center line of the stem follows by integrating Eq. (S12) subject to $x(0) = z(0) = 0$. We have solved the system numerically by implementing a shooting method in Mathematica.

B. Approximating bending stiffness. To quantify the effect of fluid redistribution from fruit to stem, and the corresponding change in orientation of the fruit, we fit the stem shape to lab images from the first time lapse image (taken approximately four days prior to launch), and the final time lapse image prior to launch (taken within 30 minutes of launch). For each image, we first fit an ellipsoid to the fruit, extracting the major and minor semi-axes, h_1 and h_2. The combined mass M of the fruit and the stem relates to the individual masses via

$$M_{tot} = M_f + M_s = \rho_f \frac{4\pi}{3} h_1 h_2^2 + \rho_s \gamma L. \qquad \text{[S26]}$$

The combined mass M_{tot} ranges from 6 to 12 g (see main text Fig 2E). Inputting a value of M_{tot}[§], an estimate of the fruit density $\rho_f (= \rho_{\text{fl}} \approx 1\,\text{g}\,\text{cm}^{-3})$, and extracting values of a, b, and γL from the image, we thus obtain a value for ρ_s. The parameters for the intrinsic curvature of the stem are assumed to stay constant through development, and come from fitting the stem model to the image of the stem post-ejection, i.e. with the fruit detached so that the stem is in its reference state[¶]. The other stem parameters, $\{r_0, m_{taper}\}$, and the base angle θ_0, are also easily extracted from the image. The only remaining unknown therefore is the Young's modulus E_s. To obtain E_s, we solve the boundary value problem and overlay the solution on the image, while varying E_s (within the Manipulate framework in Mathematica) until a best visual fit is obtained. The result is given in the main text, Fig. 2A, with parameter values for each image given in Table S1.

As noted in the main text, the redistribution of fluid causes the stem to elongate and expand, with an increase in the base radius r_0 of about 25%, and an increase in length of about 45%. The increase in radius has a strong impact on the bending stiffness, since I_s scales as the fourth power of radius. However, the increase in r_0 is not sufficient to account for the significant decrease in bending of the stem. Indeed, as reflected in the best-fit values appearing in Table S1, we found that it was necessary to increase the Young's modulus E_s by a considerable amount, a factor of almost 8.

To corroborate our approach, we also conducted experiments on a stem with the fruit detached. A cantilevered horizontal fruit-less stem was deflected by attaching fixed weights to the tip. Since the internal pressure was unchanged throughout, we anticipate being able to fit the shape under different weights for a single effective Young's modulus E_s. The result appears in Fig S3, with parameter values given in Table S2. Note that the stem shows non-trivial deflection even in Fig S3A, with no weight attached. This is due in part to self-weight, which is more significant in a horizontally cantilevered stem, but also due to intrinsic curvature separate from the bending at the tip. This is a feature we observed in some, but not all stems, and can be accounted for by changing Equation S16 to

$$\hat{u}(S) = u_0 - duS - C\exp\left(\frac{-\gamma(S-(L-dS))^2}{\sigma^2}\right), \qquad \text{[S27]}$$

where the first two terms enable for a linearly varying intrinsic curvature, and form two additional parameters that are fit from the image with no weight attached (after inputting a stem mass of 2 g and taking for

[§] We did not obtain a measure of the total mass for the time-lapsed fruit, so we have used a value of $M_{tot} = 8$ in our analysis. A different value would have a small quantitative, but not qualitative, effect on the results that follow.

[¶] Technically the stem is under the force of its own weight, but this has negligible effect on the tip curvature.

Parameter	Units	Initial	Inflated
r_0	cm	0.17	0.21
m_{taper}	-	0.01	.01
C	cm^{-1}	4	4
dS	cm	0.2	0.2
σ	cm	0.3	0.3
L	cm	3.2	3.2
γ	-	1	1.45
θ_0	radians	1.67	1.7
a	cm	1.75	1.65
b	cm	1	0.97
E_s	MPa	6	50

Table S1. Extracted parameter values for the stem model in the initial (first image of timelapse) and inflated (last image from timelapse before seed ejection) states.

simplicity uniform density due to only slight tapering). Fig S3A-D show the best fit stems for masses of 0, 4.0 g, 4.7 g, and 21.8 g attached to the ends, respectively. The different cases were all fit with the single value $E_s = 40$ MPa, a value that is consistent with those extracted from the timelapse images.

Parameter	Value
r_0	0.4 cm
m_{taper}	0.007
C	-4 cm^{-1}
dS	0.3 cm
σ	0.3 cm
u_0	0 cm^{-1}
du	0.002 cm^{-2}
E_s	40 MPa

Table S2. Parameter values corresponding to the model fits of Fig S3.

C. Fruit rotation. During the first tenth of a millisecond or so of ejection, while the stem and fruit are separating, the stem begins to rotate away from the fruit, while the fruit begins to rotate in the opposite direction at a slower rate. The rotation of the stem may be understood as a mechanical response to the combination of the impinging jet pushing against the stem and having the force and moment induced by the fruit removed. Without the fruit deforming the stem, the stem recoils away, eventually settling to a nearly straight configuration (the stem is still loaded by self-weight, though this has a much smaller effect than the load of the fruit).

As a consequence of the balance of angular momentum between the fruit and the stem, it follows that the fruit must rotate in the opposite direction. To model the rotation imparted during these first moments of ejection, we return to the balance of angular momentum of the stem, but with an added inertial term; namely, Eq. (S14) is replaced by

$$\mathbf{m}'(S,t) + \mathbf{r}'(S,t) \times \mathbf{n}(S,t) = \rho I \mathbf{d}_1 \times \ddot{\mathbf{d}}_1, \quad \text{[S28]}$$

where overdots denote time derivatives and

$$\mathbf{d}_1 = -\sin\theta \mathbf{e}_x + \cos\theta \mathbf{e}_z \quad \text{[S29]}$$

is the normal vector to the center line of the stem, following the notation in (4). The key to making progress is to localize the analysis to the first few hundred microseconds of ejection and to the tip of the stem. To this end, we define t_{sep} as the time scale associated with the separation of fruit and stem – examination of the high speed videos suggests that $t_{\text{sep}} \approx 0.1$ms. We thus restrict our analysis to the time range $0 < t < t_{\text{sep}}$, such that at $t = 0$, the system begins in the configuration corresponding to static equilibrium, and for $t > t_{\text{sep}}$, the fruit is no longer attached to the stem. During this time range, the boundary condition applied

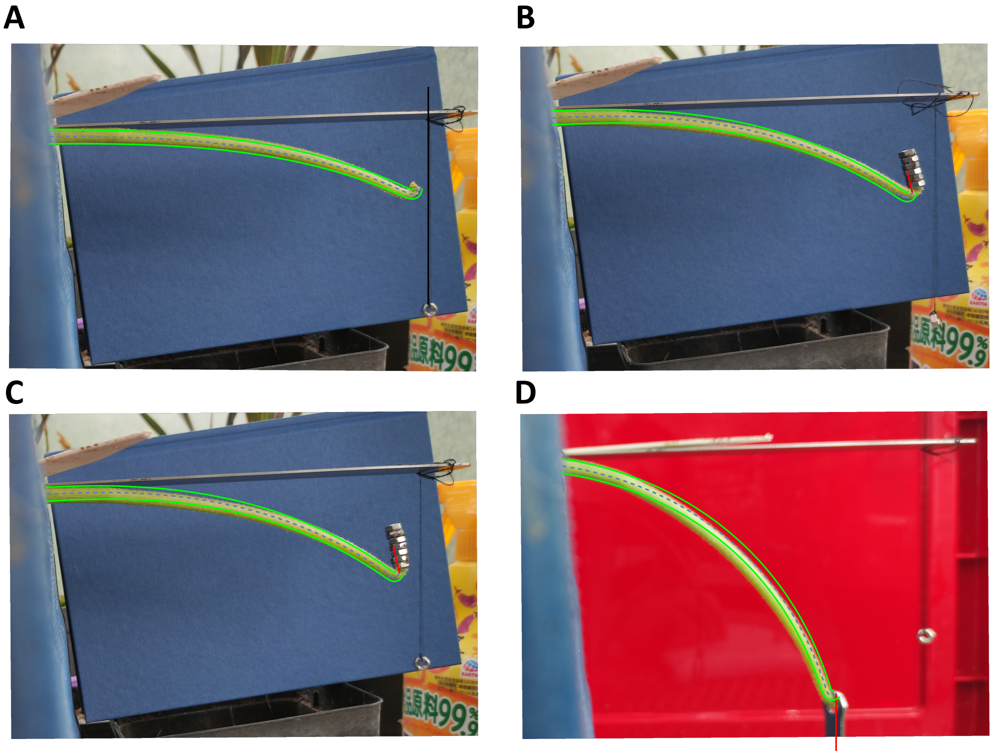

Fig. S3. The deformation of a cantilevered stem, with fruit detached, and masses of 0 (A), 4.0 g (B), 4.7 g (C), and 21.8 g (D) applied to the end. The solutions of the stem model with corresponding force and moment applied to the tip are overlaid, using parameter values shown in Table S2

by the fruit to the stem thus vanishes. Concurrently, the emerging jet pushes against the stem. We model these effects by replacing Eq. (S20) with

$$\mathbf{n}(L,t) = -M_f g f(t)\mathbf{e}_z - F_j(t)\mathbf{e}_f. \qquad \text{[S30]}$$

The first term is the same as in the static case, describing the load applied by the fruit, but with an added time dependent factor $f(t)$, a dimensionless function that has the property $f(0) = 1$, $f(t_{\text{sep}}) = 0$. We implement the form $f(t) = 1 - (t/t_{\text{sep}})^{\alpha_{\text{sep}}}$, where the constant α_{sep} characterises the separation (we use a large exponent, $\alpha_{\text{sep}} = 6$, to capture the fact that the force remains nearly constant until very close to t_{sep}). The second term describes the force of the jet on the stem. The magnitude $F_j(t)$ can be deduced from Bernoulli's relation $\Delta p = \frac{1}{2}\rho_{\text{fl}}U^2$, which relates the pressure difference across the fruit to the fluid density and velocity. We obtain a force by multiplying by the cross-sectional area, and take U over this short time scale to be equal to the initial velocity U_0, that is we fix

$$F_j \equiv \frac{1}{2}\rho_{\text{fl}}U_0^2\pi(r_0 - m_{taper}L)^2. \qquad \text{[S31]}$$

The unit vector $\mathbf{e}_f$ points along the axis of the fruit, from the center of the fruit towards the stem. At $t = 0$, we have $\mathbf{e}_f = -\mathbf{d}_3(L)$, i.e. the jet force is in the negative of the tangent direction at the tip of the stem. Considering the moment balance localized to the tip of the stem, we insert the constitutive law Eq. (S15), the geometric relation Eq. (S12), and Eq. (S30) into the moment balance Eq. (S28), giving

$$\frac{d}{dS}\left[E_s I_s(s)(\gamma^{-1}\theta'(S) + \hat{u}(S))\right]\bigg|_{S=L} - \gamma\cos\theta M_f g f(t) + \gamma\frac{1}{2}\rho_{\text{fl}}U_0^2\pi(r_0 - m_{taper}L)^2\sin\varphi(t) = \rho_s I_s\ddot{\theta}, \qquad \text{[S32]}$$

where $\varphi(t)$ is the angle between $\mathbf{d}_3$ and $-\mathbf{e}_f$ (see Fig. S4). Our approach is to integrate this equation from $t = 0$ to $t = t_{\text{sep}}$ in order to estimate $\dot{\theta}(L, t_{\text{sep}})$. Since the fruit and stem are still in contact up until t_{sep}, the

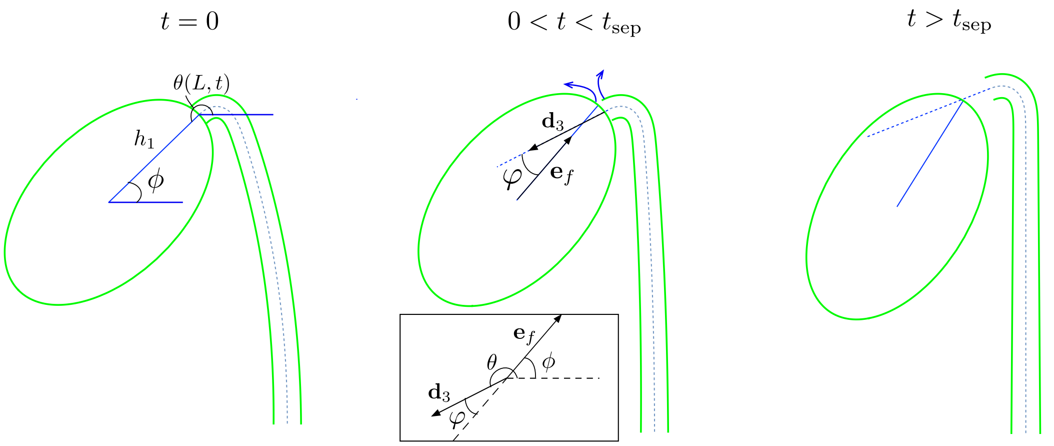

Fig. S4. Schematic of the initial stage of ejection, while the fruit and stem are separating.

fruit will counter rotate. Defining $\phi(t)$ as the angle between the fruit axis $\mathbf{e}_f$ and the horizontal direction (so , we may approximate the angular velocity of the fruit at the point of separation as

$$\dot{\phi}(t_{\mathrm{sep}}) = -\epsilon\dot{\theta}(L, t_{\mathrm{sep}}), \qquad \text{[S33]}$$

where $\epsilon = \frac{r_0 - m_{taper}L}{a}$ is the aspect ratio of the radius of the tip to the major axis of the fruit. This factor accounts for the different radii of rotation for stem and fruit.[‖] The fact that $\epsilon < 1$ implies that the fruit will have a smaller (in magnitude) angular velocity, as can be observed in high-speed videos.

In order to estimate $\dot{\theta}(L, t_{\mathrm{sep}})$, we consider the size of the different terms in Eq. (S32). Scaling time by t_{sep} and lengths by γL, we may write Eq. (S32) in non-dimensional form as

$$\ddot{\theta}(L,t) = \lambda_1\left(\gamma^{-1}\theta'(L,t) + \hat{u}(L)\right) + \lambda_2\left(\gamma^{-1}\theta''(L,t) + \hat{u}'(L)\right) - \lambda_3\gamma\cos\theta(L,t)f(t) + \lambda_4\gamma\sin\phi(t), \qquad \text{[S34]}$$

where time and space derivatives are dimensionless, and we define the dimensionless parameters

$$\lambda_1 = \frac{E_s I_s'(L) t_{\mathrm{sep}}^2}{\rho_s I_s(L)\gamma^2 L^2}, \quad \lambda_2 = \frac{E_s t_{\mathrm{sep}}^2}{\rho_s \gamma^2 L^2}, \quad \lambda_3 = \frac{M_f g t_{\mathrm{sep}}^2}{\rho_s I_s(L)}, \quad \lambda_4 = \frac{\rho_{\mathrm{fl}} U_0^2 \pi (r_0 - m_{taper}L)^2 t_{\mathrm{sep}}^2}{\rho_s I_s(L)}. \qquad \text{[S35]}$$

Typical values for the inflated stem are $E_s \sim 50\mathrm{MPa}$, $I_s(L) \sim 10^{-3}\mathrm{cm}^4$, $I_S'(L) \sim 10^{-4}\mathrm{cm}^3$, $L \sim 5\mathrm{cm}$, $\gamma \sim 1.5$, $\rho_s \sim 1\mathrm{g/cm}^3$, and $r_0 - m_{taper}L \sim 0.2\mathrm{cm}$. For the fruit, $M_f \sim 5\mathrm{g}$, and the fluid has initial fluid velocity $U_0 \sim 2\mathrm{cm/ms}$ and density $\rho_{\mathrm{fl}} \sim 1\mathrm{g/cm}^3$. Inserting these values, along with $t_{\mathrm{sep}} \sim 0.1\mathrm{ms}$, we estimate

$$\lambda_1 \sim 0.01, \quad \lambda_2 \sim 0.1, \quad \lambda_3 \sim 0.05, \quad \lambda_4 \sim 5. \qquad \text{[S36]}$$

This analysis suggests that the first three terms can be taken to be approximately constant during the separation phase. Despite λ_4 being order 1, the fourth term is also small, because the fruit and stem remain connected during $0 < t < t_{\mathrm{sep}}$, and thus the axis of the fruit and the tangent to the tip of the stem are very nearly aligned. Since $\varphi(0) = 0$, we may approximate $\varphi(t) \approx t^{\beta_{\mathrm{sep}}}$ (φ is exaggerated in the schematic Fig S4) for some $\beta_{\mathrm{sep}} \geq 1$[**]. Defining $\theta_1(t) := \theta(L,t)$, we may thus approximate

$$\ddot{\theta_1}(t) = \lambda_1\left(\gamma^{-1}\theta'(L,0) + \hat{u}(L)\right) + \lambda_2\left(\gamma^{-1}\theta''(L,0) + \hat{u}'(L)\right) - \lambda_3\gamma\cos\theta(L,0)f(t) + \lambda_4\gamma t^{\beta_{\mathrm{sep}}}, \qquad \text{[S37]}$$

[‖] The situation is comparable to two balls of different radii that are in contact: if the smaller ball rotates and 'drags' the other ball to counter-rotate, the angular velocities will be of opposite sign and with constant of proportionality equal to the ratio of the radii, with the smaller ball having a higher angular velocity.

[**] The best choice for β_{sep} will depend on the nature of the dehiscence process and any torque-inducing asymmetry of the emerging jet. Such details are beyond the scope of this work, so we take β_{sep} as a free parameter obtained from experimental observations; we then keep the same value of β_{sep} in the subsequent comparative analysis when changing other parameters.

where the values of θ and its derivatives evaluated at $S = L$ and $t = 0$ come from the equilibrium solution. We may now integrate this equation explicitly from $t = 0$ to $t = t_{\text{sep}}$, giving

$$\dot{\theta}_1(t_{\text{sep}}) = t_{\text{sep}} \left[\lambda_1 \left(\gamma^{-1}\theta'(L,0) + \hat{u}(L) \right) + \lambda_2 \left(\gamma^{-1}\theta''(L,0) + \hat{u}'(L) \right) \right] - \quad \text{[S38]}$$

$$\lambda_3 \gamma \cos\theta(L,0) \int_0^{t_{\text{sep}}} f(t)\, dt + \lambda_4 \gamma \frac{t_{\text{sep}}^{\beta_{\text{sep}}+1}}{\beta_{\text{sep}}+1} \quad \text{[S39]}$$

where we have used $\dot{\theta}_1(0) = 0$.

Once the fruit is detached from the stem, it falls under gravity and rotates due to the initial angular velocity provided by the stem during detachment, but with no further applied torque (due to the approximate symmetry of the jet, the reaction force does not generate a torque about the center of mass). Thus, for $t > t_{\text{sep}}$, the balance of angular momentum for the fruit reads

$$\frac{d}{dt}\left(I_f(t)\dot{\phi}(t) \right) = 0, \; \phi(t_{\text{sep}}) = \theta(L,0) - \pi, \; \dot{\phi}(t_{\text{sep}}) = -\epsilon \dot{\theta}_1(t_{\text{sep}}). \quad \text{[S40]}$$

Since the fruit is losing mass during ejection, the moment of inertia I_f is a function of time. Recalling that we model the fruit as an ellipsoid with major semi-axis a and both minor semi-axes b, and that I_f is the moment of inertia for rotation about one of the minor axes, I_f can be computed to be

$$I_f = \frac{M_f}{5}\left(a^2 + b^2 \right). \quad \text{[S41]}$$

To see how I_f varies in time, and to formulate the components needed for computation of seed trajectories, we now turn to a model of the ejection of the fluid/seeds.

D. Fluid/seed ejection. As stated above, the jet velocity $U(t)$ (taken to be spatially homogeneous) relates to the pressure difference Δp via Bernoulli's relation:

$$\Delta p = \frac{1}{2}\rho_{\text{fl}} U^2. \quad \text{[S42]}$$

(Note that we assume that the seeds move with the instantaneous speed of the liquid jet here; however, a balance between the work done by the pressure and the kinetic energy of the seed gives the same result for the launch speed, as shown for fungal spores (6).) The pressure difference is also related to the typical tension, $\bar{\sigma}$, in the wall of the fruit via the Young–Laplace law

$$\Delta p = 2\bar{\sigma} K^{1/2}, \quad \text{[S43]}$$

where $K = (R_x R_y)^{-1}$ is the Gaussian curvature of the surface, and $R_x = a^2/b$, $R_y = b$ are the radii of curvature of the shell. The typical tension $\bar{\sigma}$ is equal to the product of the stretching modulus, Y, and the typical strain $\bar{\varepsilon}$. The modulus Y is the product of the Young's modulus E_f and the fruit wall thickness, which we denote h, while the strain can be expressed in terms of the change in volume $\Delta V = V(t) - V_{\text{nat}}$ between the current volume and the volume of the shell in its natural state. Specifically,

$$\bar{\varepsilon} = \frac{1}{3}\frac{\Delta V}{V_{\text{nat}}}. \quad \text{[S44]}$$

By combining Eq. (S42)–Eq. (S44), we therefore have that the instantaneous velocity of ejected fluid is a function of the volume of the shell via

$$U = (2\Delta p/\rho_{\text{fl}})^{1/2} = \frac{2U_{EI}}{\sqrt{3}}\left(\frac{V(t) - V_{\text{nat}}}{V_{\text{nat}}} \right)^{1/2}, \quad \text{[S45]}$$

where

$$U_{EI} = \left(\frac{Y}{\rho_{\text{fl}} a(0)}\right)^{1/2}$$

is an 'elasto-inertial speed'. Using the value $Y \approx 2$ kN/m (see discussion after Eq. (S10)), $\rho_{\text{fl}} \approx 1000\ \text{kg}\,\text{m}^{-3}$ and $a(0) \approx 2$ cm we have that $U_{EI} \approx 10\ \text{m}\,\text{s}^{-1}$.

To determine the evolution of the volume $V(t)$, we then use conservation of mass, which may be written

$$\frac{\text{d}V}{\text{d}t} = -A_{\text{jet}} U, \qquad \text{[S46]}$$

with A_{jet} the cross-sectional area of the jet. For our simulations, we take A_{jet} to be equal to 90% of the area of the tip of the stem, $E_f = 0.64$ MPa, and $h = 0.32$ cm, in accordance with the measurements outlined in the main text. Combining the above equations, we obtain a differential equation for $V(t)$,

$$\frac{\text{d}V}{\text{d}t} = -\frac{2A_{\text{jet}} U_{EI}}{\sqrt{3}} \sqrt{\frac{V(t) - V_{\text{nat}}}{V_{\text{nat}}}}, \qquad \text{[S47]}$$

which can be solved exactly subject to the initial condition[††] $V(0) = V_0$ to give

$$V(t) = V_{\text{nat}} + \left(\sqrt{V_0 - V_{\text{nat}}} - \frac{2}{\sqrt{3}} \frac{U_{EI} A}{\sqrt{V_{\text{nat}}}} t\right)^2, \qquad \text{[S48]}$$

and

$$U(t) = \frac{2U_{EI}}{\sqrt{3}} \sqrt{\frac{V_0 - V_{\text{nat}}}{V_{\text{nat}}}} - \frac{4}{3} U_{EI}^2 \frac{A}{V_{\text{nat}}} t. \qquad \text{[S49]}$$

Note that the initial (and maximum) velocity, depends on the volume difference between the pressurized and natural states; we take $V_0 := \frac{4\pi}{3} a(0) b(0)^2$, with the values of $a(0)$ and $b(0)$ extracted from the final experimental image before ejection. Based on our measurements of mass loss before and after ejection, we estimate that the volume in the natural state is 20 to 50% less than the volume at the point of dehiscence. In our simulations, we have used the value $V_{\text{nat}} = 0.45 V_0$. This means that the initial mean strain, $\bar{\epsilon}(0) \approx 1/3$, which is not strictly small. Nevertheless, the model agrees remarkably well with experimental observations.

Returning to the balance of angular momentum of the fruit, Eq. (S40), we incorporate the loss of mass during ejection by substituting the expression for $V(t)$ in the relation $M_f = \rho_{\text{fl}} V(t)$. The major and minor axes $a(t)$ and $b(t)$ will also decrease during ejection. Recalling the relation $V = \frac{4\pi}{3} ab^2$, we can account for this by making the assumption that the ratio between a and b remains roughly constant while the fruit deflates. Defining $\xi = b(0)/a(0)$ as this ratio leads to the equation

$$4\pi \xi^2 a(t)^2 \frac{\text{d}b}{\text{d}t} = \frac{\text{d}V}{\text{d}t}, \qquad \text{[S50]}$$

which can be solved for a, thereby giving an explicit expression for the moment of inertia $I_f(t)$ defined in Eq. (S41).

E. Ballistic trajectories. The ballistic trajectories $\mathbf{r}(t)$ of individual seeds of mass m are computed by integrating forward the kinematic relation

$$m\ddot{\mathbf{r}}(t) = -mg\mathbf{e}_z - F_d(|\dot{\mathbf{r}}(t)|) \frac{\dot{\mathbf{r}}(t)}{|\dot{\mathbf{r}}(t)|}, \qquad \text{[S51]}$$

where g is the gravitational acceleration, acting in the vertical direction $\mathbf{e}_z$. These calculations account for air resistance by considering the drag force acting on individual seeds to be given by $F_d(U) =$

[††] Note that we have shifted the ejection time to start at $t = 0$ as opposed to $t = t_{\text{sep}}$, which can be done without issue as this modeling component is decoupled from the balance of angular momentum during $0 < t < t_{\text{sep}}$ outlined above.

$(1/2)\rho_{air}C_dU^2A_{\rm se}$ where $A_{\rm se} = \pi w_{\rm se}t_{\rm se}/4 \approx 5\,{\rm mm}^2$ is the cross-sectional area of the seed normal to the flow direction, $w_{\rm se}$ and $t_{\rm se}$ are the widths and thicknesses of seeds, respectively, and the coefficient of drag, $C_d = (24/Re_{air}) + 6/(1 + Re_{air}^{1/2}) + 0.4 = 0.4$, is related to the Reynolds number of seeds in air, $Re_{air} = \rho_{air}w_{\rm se}U/\mu_{air} \sim 5\times10^6 \gg 1$ where the width of a seed $w_{\rm se} \approx 3\,{\rm mm}$, and the density and dynamic viscosity of air are $\rho_{\rm air} \approx 1.3\ {\rm kg\,m^{-3}}$ and $\mu_{\rm air} \sim 10^{-5}\,{\rm Pa\,s}$, respectively.

E.1. Optimal launch angle. One question that immediately arises from the Eq. (S51) is how the drag force F_d changes the ballistic trajectory of the seed, and whether the furthest reach is still attained with an initial launch angle $\phi = 45°$? Non-dimensionalization of Eq. (S51) reveals a dimensionless parameter

$$\mathcal{G} = \frac{m_{\rm se}g}{\frac{1}{2}\rho_{\rm air}C_dU_0^2A_{\rm se}} \qquad \text{[S52]}$$

that compares the weight of the seed, $m_{\rm se}g$, to the drag force $F_d(U_0)$ on the seed at its initial speed, U_0.

For a given value of $\mathcal{G}$, we compute the trajectories of projectiles with a range of launch angles and compute the horizontal range, $x_{\rm range}(\phi) = x(z = 0;\phi)$. As expected, we see that as the angle of launch, ϕ, varies $x_{\rm range}(\phi)$ is maximized at a particular value of ϕ, $\phi_{\rm opt}$; in this way, it is possible to determine the optimal launch angle, $\phi_{\rm opt}(\mathcal{G})$, numerically. A plot of this function is given in fig. S5, and shows that the optimal angle lies a few degrees below the classic value of 45° unless $\mathcal{G} \gtrsim 10$. Using estimates of the seed parameters $m_{\rm se} \approx 25$ mg, $C_d = 0.4$ and $5\ {\rm ms}^{-1} \le U_0 \le 25\ {\rm ms}^{-1}$ together with $A_{\rm se} \approx 5\ {\rm mm}^2$, we find that $0.4 \lesssim \mathcal{G} \lesssim 10$, which is shown as the blue square in fig. S5. For this range of $\mathcal{G}$, $36.9° \lesssim \phi_{\rm opt} \lesssim 44.4°$, which is consistent with the observed launch angles $\phi \approx 42.7 \pm 8.9°$ being close to optimal (main text).

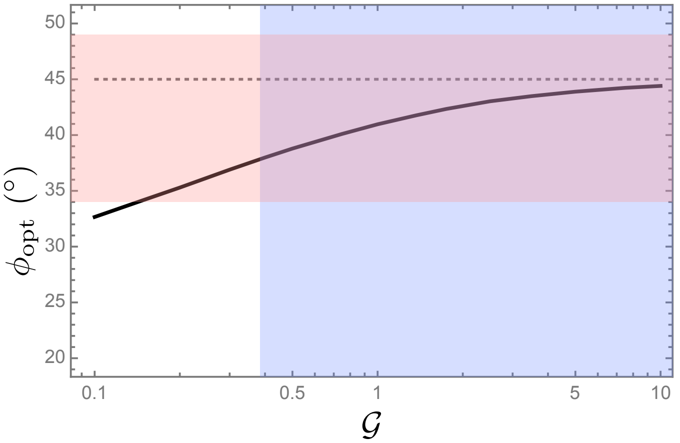

Fig. S5. The optimal launch angle, $\phi_{\rm opt}$, as a function of the projectile weight to maximum drag, $\mathcal{G}$, defined in Eq. (S52). Numerically determined value of $\phi_{\rm opt}$ (solid curve) tends to the drag-free result $\phi_{\rm opt} = 45°$ (dashed line) only in the limit $\mathcal{G} \to \infty$. The experimentally observed range of $\mathcal{G}$ is indicated by the blue rectangle; the experimentally-observed values of ϕ are indicated by the red rectangle; the overlap between the two (purple rectangle) lies close to the curve of optimal angles.

E.2. Trajectories of multiple seeds. Evaluating U given by Eq. (S49) at discrete time points during ejection gives the magnitude of the initial velocity vector of individual seeds. The direction of the initial velocity vector comes from integrating the system Eq. (S40) and evaluating the angle of the fruit axis $\phi(t)$ at the same discrete time points. Rather than divide the ejection time in equally spaced units, it is more accurate to suppose that equal volumes of ejecting fluid will contain approximately equal numbers of seeds. Thus, the discrete time points are obtained by dividing the excess volume $\Delta V = V - V_{\rm nat}$ into N equal increments, $\Delta V_i = \frac{i}{N}(V_0 - V_{\rm nat})$ where N is the total number of seeds in the fruit. Note that not all seeds will be ejected; since the ejection speed decreases with time, the fluid velocity may not be high enough to eject seeds located away from the fruit aperture (indeed, inspection of an exploded fruit shows that some seeds remain). The ejection time t_i then comes from solving Eq. (S48) for t with $V = V_{\rm nat} + \Delta V_i$. We account for

later seeds not leaving the fruit by only integrating the trajectories of the $N-k$ seeds; i.e. the last k seeds (corresponding to small ΔV and thus a smaller velocity) are assumed to remain in the fruit.

F. Plant generations. To simulate seed dispersal and reproduction over several generations, we perform the following steps:

1. Compute the deformation of the stem in the pressurised state, following Section A.
2. Compute the rotation of the fruit during detachment following Section C.
3. Compute initial velocities for ejected seeds following Section D.
4. Compute the ballistic trajectories and landing location of expelled seeds by integrating forward Eq. (S51) until $z(t) = \mathbf{r} \cdot \mathbf{e}_z = 0$. This is repeated for each fruit on the plant. The result is a distribution of seeds surrounding the mother plant.
5. Determine which seeds will germinate and produce a next generation plant, by applying a probabilistic rule and minimum distance from successful offspring.
6. For each successful plant, return to Step 4 and repeat the procedure for each successful plant to simulate the next generation.

More on Step 3 To simulate dispersal from all fruits of a given plant, we first assign a number M of fruits per plant. In our simulations, we have used $M = 30$ fruits per plant, and $N = 30$ seeds ejected per fruit, so that each plant ejects $N * M = 900$ seeds. We position each of the fruits for a given plant at the same location, the location of the plant; e.g. the first plant is located at the origin of our coordinate system, $\mathbf{r} = \mathbf{0}$, and all fruits of the first plant are positioned at the same location. This assumption simplifies calculations, and may be justified by the separation in length scale between the width of an individual plant, on the order of centimeters, and the typical dispersal distance of a seed, 3 m.

The initial velocity for each individual fruit is obtained by rotating the initial velocity vector described in Section E about the vertical, i.e. the z axis, by an angle that is drawn from a uniform $[0, 2\pi]$ radians distribution. This introduces a non-deterministic component to the simulations, and reflects the observation that the fruit locations do not show a clear pattern or bias in orientation around the center of the plant.

We also add a second stochastic component to the initial velocity of individual seeds. By construction, the model is two-dimensional, and thus the deterministic flight path of the seeds lies in a plane. Simulating seed dispersal in this way produces an unrealistic distribution in which seeds land in radial lines emanating from each fruit. In reality, the interaction between the seeds and the fluid jet as well as other inherent symmetry-breaking noise in the system creates a slight angular dispersal in the flight paths. To incorporate this in our simulation, we apply a slight additional rotation about the vertical axis to each individual seed, with rotation angle for each seed drawn from a uniform $[-0.2, 0.2]$ radians distribution.

More on Step 5 The germination of any given seed will depend on numerous factors, including soil quality, water availability, and environmental conditions. If a seed germinates to a seedling, the survival of a seedling is also dependent on many of the same factors, but also involves a competition for space: a seedling that is too close in proximity to another plant or another successful seedling is unlikely to survive. Our objective here is not to describe or model this complex process, but rather to investigate qualitatively how the input at the level of the stem and fruit may contribute to the success over several generations. Thus, we take a black box approach in which each seed is chosen to produce a successful plant at the next generation based on assigning a fixed probability of success, but conditioned on a proximity requirement. In the simulations shown in Figure 4of the main text, we have implemented a probability of survival of $p = 1.5\%$, as well as a requirement for success that a seed is greater than or equal to $\delta_{\min} = 2.5$ m from any other successful seedling and/or existing plant. These particular values were not chosen based on quantitative data, for such

data does not, to our knowledge, exist for *Ecballium*. Naturally, different choices will have a quantitative effect on reproductive success, e.g. how many new plants appear in the third generation. To demonstrate that the same qualitative features appear for different input parameters, we show in Fig S6 the results of simulations with increased and decreased germination probabilities and minimum distance threshold. The top row, $p = .015$, $\delta_{\min} = 2.5$ m, corresponds to the values used in Main text Fig 4. The second row shows results with a decreased probability and increased minimum distance, which causes the number of successful plants at the third generation to be diminished. The third row represents an unrealistic regime in which the probability is increased and the minimum distance decreased, leading to a significant increase in third generation plants. The fourth row shows results for an increased probability and increased threshold distance. For each set of p and $\delta_{\min}$, the qualitative features across the different fruit/stem types are similar, and the plant with the baseline parameters determined from laboratory measurements of real specimens generates in each case the most plants at third generation.

G. Comparative analysis parameter changes. In simulating the three hypothesized mutant plants, we have made the following parameter changes from the base case:

1. **Under-pressurised stem** Inflated stem radius at base reduced from $r_0 = 0.21$ cm to $r_0 = 0.19$ cm; inflated effective Young's modulus of stem reduced from $E_s = 50$ MPa to $E_s = 10$ MPa. In inflated state, fruit major axis increased from $a = 1.65$ cm to $a = 1.7$ cm; minor axis increased from $b = 0.97$ cm to $b = 0.99$ cm.

2. **Stiffer stem** Stem radius at base initial state increased from $r_0 = 0.17$ cm to $r_0 = 0.3$ cm; the same degree of inflation was applied as the base case, so that the stem radius in the inflated state was $r_0 = 0.37$. The stem intrinsic curvature for this was also decreased in magnitude, with the parameter C changed from $C = -4$ cm^{-1} to $C = -3.5$ cm^{-1}. This change was made so that a stiff stem would result in a nearly horizontal fruit.

3. **Over-pressurised stem** In the initial state, the fruit major axis was increased from $a = 1.75$ cm to $a = 2.0$ cm; and the minor axis was increased from $b = 0.97$ cm to $b = 1.1$ cm. The same decrease during stem pressurisation was applied as in the base case, so that in the pressurised (stem) state, the major axis was $a = 1.88$ cm (compared to $a = 1.65$ cm in base case), and the minor axis was $b = 1.07$ cm ($b = 0.97$ cm in base case). The total mass of fruit and stem was also increased from $M_{tot} = 9$ g to $M_{tot} = 9.5$ g.

H. Fluid redistribution. Our comparative analysis demonstrates that successful dispersal requires a launch angle close to 45 degrees, sufficient fruit pressure to generate high seed velocities, and sufficient but not excessive fruit rotation to create a good distribution of landing locations. Each of these components depends on numerous geometric and mechanical parameters, as described through each level of our mathematical model. The full parameter space is high dimensional – e.g. there are 11 parameters in the properties of the stem and fruit (as given in Table S1), plus additional parameters characterizing the pressurization of the fruit, properties of the seeds, density of the mucilaginous fluid, etc. It is impractical to independently explore the sensitivity of the dispersal mechanism to all model parameters. Moreover, several key parameters are linked, e.g. the stiffness of the stem depends on the geometric dimensions as well as the degree of stem inflation. Still, the question remains to what degree *Ecballium* has been bio-engineered by evolution to achieve successful dispersal. To provide a partial answer and in a self-consistent manner, in main text Fig 5 we have presented the sensitivity of the mean dispersal distance and number of 3rd generation plants to a critical component: fluid redistribution from fruit to stem in the days prior to launch. Here we provide the details on this calculation and further analysis.

We first recall Table S1, which lists parameter values for stem and fruit extracted before and after the redistribution of fluid. In particular, this Table shows the degree to which the fruit contracts (decrease in major axis a and minor axis b), the stem inflates (increase in base radius r_0) and elongates (increase in γ),

and the stem increases in effective stiffness E_s. To explore the impact of a greater or lesser degree of fluid redistribution, we assume that each of these parameters varies in a continuous manner based on the degree of redistribution. In particular, we define the dimensionless parameter β such that each of the parameters $\{a, b, r_0, \gamma, E_s\}$ vary linearly from the extracted initial value when $\beta = 0$ to the extracted final value when $\beta = 1$. That is, we define

$$r_0(\beta) = r_{0_{\text{init}}}(1-\beta) + r_{0_{\text{final}}}\beta \quad \text{[S53]}$$
$$a(\beta) = a_{\text{init}}(1-\beta) + a_{\text{final}}\beta \quad \text{[S54]}$$
$$b(\beta) = b_{\text{init}}(1-\beta) + b_{\text{final}}\beta \quad \text{[S55]}$$
$$\gamma(\beta) = \gamma_{\text{init}}(1-\beta) + \gamma_{\text{final}}\beta \quad \text{[S56]}$$
$$E_s(\beta) = E_{s_{\text{init}}}(1-\beta) + E_{s_{\text{final}}}\beta \quad \text{[S57]}$$

where the initial and final values correspond to those in the third and fourth columns of Table S1. Note that in this modeling framework, the pressure within the fruit will be a decreasing function of β. This is because the pressure is related to the fruit dimensions a and b via Equations S43 and S44, which combine to give

$$\Delta p = \frac{2E_f h}{3(ab)^{1/2}} \left(\frac{\frac{4}{3}\pi a b^2 - V_{\text{nat}}}{V_{\text{nat}}} \right). \quad \text{[S58]}$$

Since a and b both decrease with β, Δp does as well. A decreasing pressure would imply a decreasing fruit stiffness, according to the analysis in Sec A.2. On the other hand, our indentation data on fruits, main text Fig 2B, shows a nearly constant stiffness under decreasing fruit volume in the days building up to launch. This could be accounted for by a corresponding increase in the Young's modulus of the fruit wall during fluid redistribution, though ascertaining such a change would require a cellular-level analysis that goes beyond the scope of this work. Therefore, lacking a detailed cellular-level understanding of the redistribution process and any potential remodeling in the fruit wall, in the analysis presented below and in main text Fig 5, we have taken the simplest assumption of a constant fruit Young's modulus. Since the pressure dictates the maximum seed velocity, an alternative modeling assumption could be to keep the maximum velocity fixed for varying β. However, since the impact of β on stem stiffness and thus launch angle and fruit rotation remains the same, we find that for such an assumption, the qualitative characteristics of the analysis presented below and in main text Fig 5 do not change significantly (results not shown).

For a given value of β, we simulate the seed ballistics for seeds from a single fruit, from which we extract the following metrics: mean seed distance, maximum seed velocity, maximum seed distance, minimum seed distance, fruit angle at launch, maximum fruit rotation, and the standard deviation of the distribution of seeds. Note that in this formulation, $\beta = 1$ is the degree of fluid redistribution we have observed in the laboratory, $\beta < 1$ corresponds to seed launch occurring after less fluid redistribution ($\beta = 0$ corresponds to no fluid redistribution), and $\beta > 1$ implies a greater degree of fluid redistribution. Treating β as a continuous variable thus enables to examine the dispersal characteristics in a manner consistent with the physics underlying our model.

The metrics listed above are plotted as a function of β, with β ranging from 0 to 2, in Fig. S7A-G. As well as extracting metrics from the trajectories of seeds from a single fruit, we also simulated on a coarser grid seed dispersal over two generations, following the probabilistic model outlined above with minimum distance required 2.5 m and probability of germination $p = 0.005$. Fig. S7H plots the mean of the number of plants predicted at the 3rd generation from 100 simulations for each value of β, with error bars showing the standard deviation.

Fig. S7A plots the mean seed distance; this plot is combined with H in main text Fig. 5. Fig. S7B plots the maximum seed velocity, which is the exit velocity of the first seed. This value decreases monotonically with β, because an increase in fluid redistribution corresponds to a decrease in pressure within the fruit. This does not, however, necessarily generate better dispersal. For very small values of β, the launch angle

is close to vertical (Fig. S7E), and the fruit rotation during launch is very low (Fig. S7F) – this is because the tangent to the stem at the tip is closely aligned with gravity, therefore the moment applied by the fruit is relatively small. In this case, seeds fire mostly vertically, reaching a low maximum distance (Fig. S7C), and low standard variation (Fig. S7G). The result is that the seeds are largely clustered close together at an intermediate distance, which does not translate to generational success.

For β around 0.5, the situation is qualitatively different. The relative fluid redistribution is only half of what we observed, but is still sufficient to create a good launch angle. However, the change in angle combined with the weak stem and relatively more massive fruit means that the torque on the stem is high, creating a very large fruit rotation upon dehiscence. The result is that though some seeds reach high distances (Fig. S7C shows a max around $\beta = 0.4$), excessive fruit rotation causes other seeds to land very close to the original plant, so that the minimum seed distance is close to zero (Fig. S7D). Overall, though a good spread of seeds is attained, seeds landing too close to the mother plant have no chance to succeed, and the number of predicted plants at 3rd generation is a bit below the maximum simulated.

On the other end, these metrics also demonstrate clearly why a degree of fluid redistribution greater than we have observed, i.e. values of β greater than one, may also be detrimental to dispersal. For large β, the fruit has depressurized to a greater extent, leading to a lower maximum velocity. The stem is also stiffer, and the fruit is less massive, thus there is less torque creating less fruit rotation. The stiffness also impacts the launch angle, though as the stem has a natural curvature, the launch angle is close to constant for $\beta \gtrapprox 1$. Overall, the metrics for $\beta > 1$ are similar to those at $\beta \approx 1$, just with a decreased distance due to the decreased pressure, and thus there is no benefit to the additional redistribution, which is reflected by a decreasing number of plants at 3rd generation with increasing $\beta > 1$.

3. SI video legends

1. Video S1: CT Scan of fruit, providing detailed view of internal structure, including seed placement, size, and shell thickness.

2. Video S2: Timelapse of the final days of development of a ripe fruit. Near ripe fruits were extracted from the Oxford Botanic Garden, stem intact, and placed in a supporting tube with water. Images were acquired every 30 minutes, continuing until the fruit ejected its seeds.

3. Video S3: seed ejection filmed at 8600 frames per second. A nearly ripe fruit was extracted from the Oxford Botanic Garden, stem intact, and placed in a supporting tube with water. A high-speed camera remained aimed at the fruit for several days, with an image-based auto-trigger set up, so that seed ejection could be captured when it occurred naturally.

References

1. Vella D, Ajdari A, Vaziri A, Boudaoud A (2012) The indentation of pressurized elastic shells: from polymeric capsules to yeast cells. *Journal of The Royal Society Interface* 9(68):448–455.
2. Sun W, Paulose J (2021) Indentation responses of pressurized ellipsoidal and cylindrical elastic shells: Insights from shallow-shell theory. *Phys. Rev. E* 104(2):025004.
3. Obaton MF (1947) Sur la projection des graines de l'ecballium elaterium rich. *Bull. Soc. Bot. France* 94(3-4):95–98.
4. Moulton D, Lessinnes T, Goriely A (2013) Morphoelastic rods. part i: A single growing elastic rod. *Journal of the Mechanics and Physics of Solids* 61(2):398–427.
5. Goriely A (2017) *The Mathematics and Mechanics of Biological Growth.* (Springer Verlag, New York).
6. Roper M, Pepper RE, Brenner MP, Pringle A (2008) Explosively launched spores of ascomycete fungi have drag-minimizing shapes. *Proc. Natl Acad. Sci. USA* 105:20583–20588.

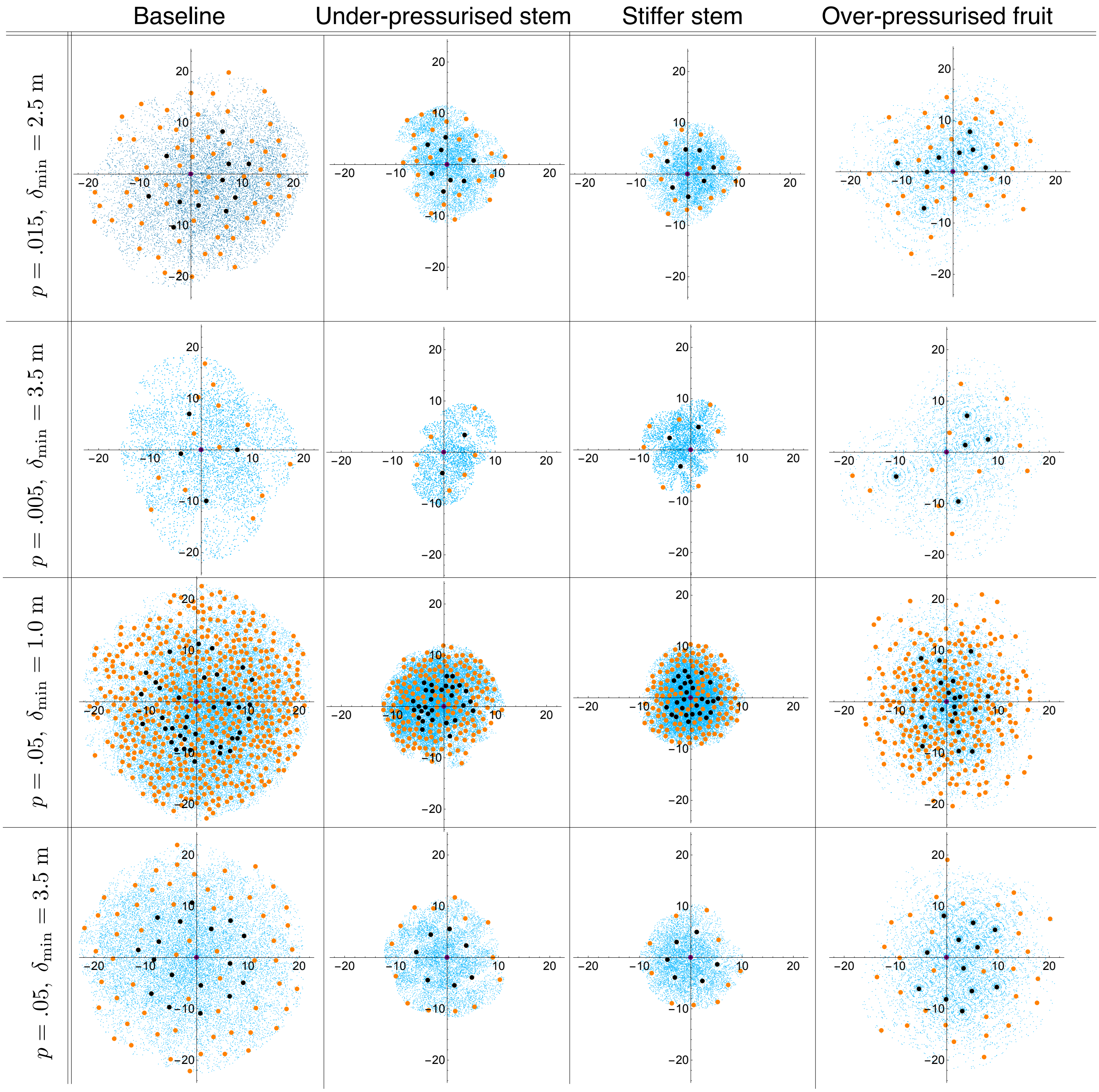

Fig. S6. Simulations of plant reproductive success over three generations, varying both input model parameters as outlined in the Comparative analysis in Main text Fig 4, and also varying the probability p that a given seed successfully produces a new plant, as well as the minimum distance δ_{min} that a seed must be from all other successful seeds and/or plants in order to survive; units in m.

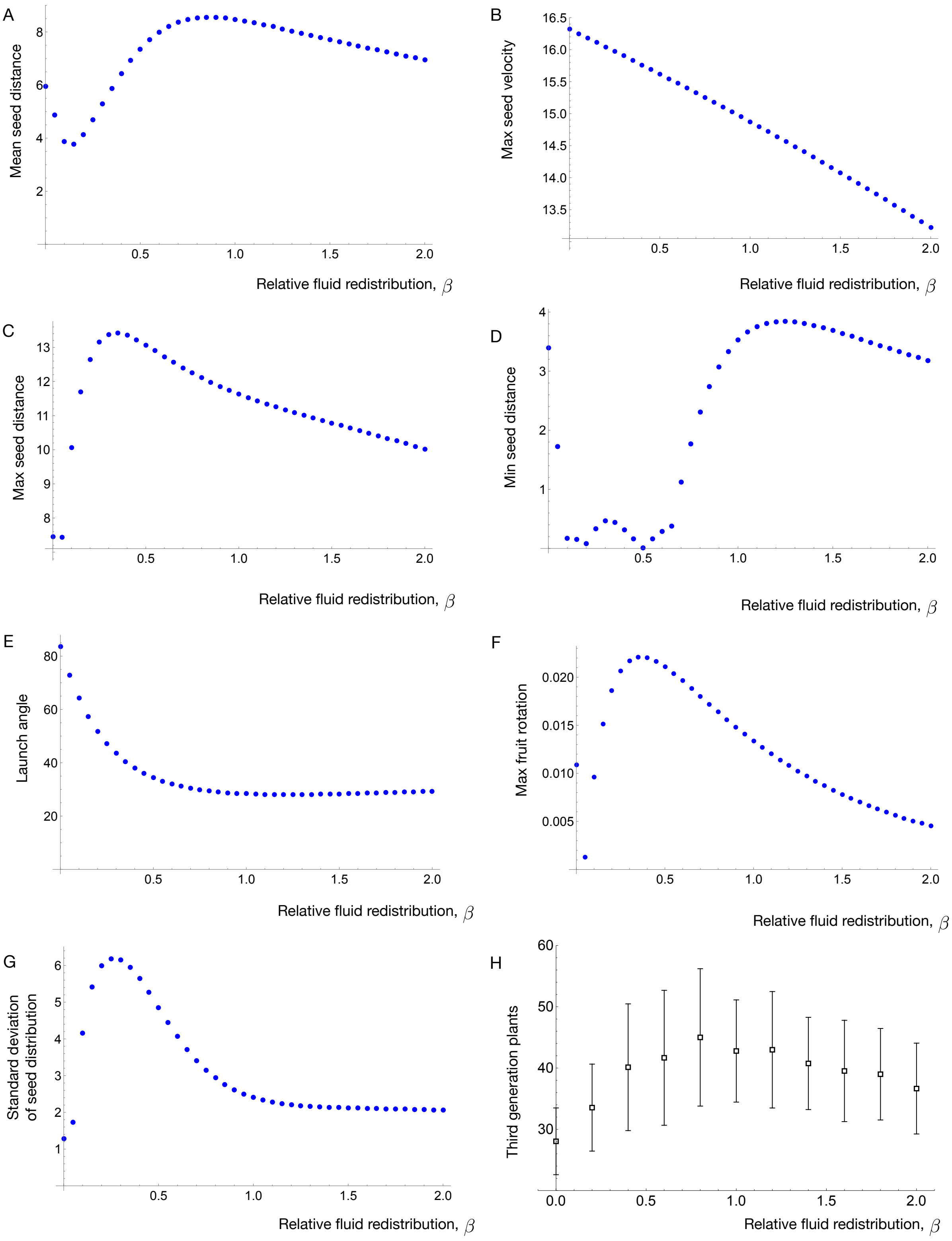

Fig. S7. Results of our analysis of fluid redistribution. In A-G, we plot the mean seed distance (A), maximum seed velocity (B), maximum seed distance (C), minimum seed distance (D), initial launch angle (E), maximum fruit rotation (in radians per ms)(F), and standard deviation of seed distribution (G), each as functions of β, computed from seed dispersal of a single fruit. We also performed 100 simulations of the probabilistic generational model for each value of β, and plot in H the mean number of 3rd generation plants, with error bars displaying the standard deviation.
